# Combining network pharmacology, machine learning, molecular docking and molecular dynamic to explore the mechanism of Chufeng Qingpi decoction in treating schistosomiasis

**DOI:** 10.3389/fcimb.2024.1453529

**Published:** 2024-09-06

**Authors:** Minglu Liu, Yuxin Wang, Wen Deng, Jiahao Xie, Yanyao He, Liang Wang, Jianbin Zhang, Ming Cui

**Affiliations:** ^1^ Emergency Department, The Second Hospital of Dalian Medical University, Dalian, Liaoning, China; ^2^ Research and Teaching Department of Comparative Medicine, Dalian Medical University, Dalian, Liaoning, China; ^3^ College of Pharmacy, Dalian Medical University, Dalian, Liaoning, China

**Keywords:** schistosomiasis, network pharmacology, molecular docking, machine learning, molecular dynamic simulation, Chufeng Qingpi decoction

## Abstract

**Background:**

Although the Chufeng Qingpi Decoction (CQD) has demonstrated clinical effectiveness in the treatment of schistosomiasis, the precise active components and the underlying mechanisms of its therapeutic action remain elusive. To achieve a profound comprehension, we incorporate network pharmacology, bioinformatics analysis, molecular docking, and molecular dynamics simulations as investigative methodologies within our research framework.

**Method:**

Utilizing TCMSP and UniProt, we identified formula components and targets. Cytoscape 3.10.0 was used to construct an herb–target interaction network. Genecards, DisGeNET, and OMIM databases were examined for disease-related objectives. A Venn diagram identified the intersection of compound and disease targets. Using Draw Venn, overlapping targets populated STRING for PPI network. CytoNCA identified schistosomiasis treatment targets. GO & KEGG enrichment analysis followed High-scoring genes in PPI were analyzed by LASSO, RF, SVM-RFE. Molecular docking & simulations investigated target-compound interactions.

**Result:**

The component’s target network encompassed 379 nodes, 1629 edges, highlighting compounds such as wogonin, kaempferol, luteolin, and quercetin. Amongst the proteins within the PPI network, PTGS2, TNF, TGFB1, BCL2, TP53, IL10, JUN, MMP2, IL1B, and MYC stood out as the most prevalent entities. GO and KEGG revealed that mainly involved the responses to UV, positive regulation of cell migration and motility. The signal pathways encompassed Pathways in cancer, Lipid and atherosclerosis, Fluid shear stress and atherosclerosis, as well as the AGE-RAGE. Bioinformatics analysis indicated TP53 was the core gene. Ultimately, the molecular docking revealed that wogonin, kaempferol, luteolin, and quercetin each exhibited significant affinity in their respective interactions with TP53. Notably, kaempferol exhibited the lowest binding energy, indicating a highly stable interaction with TP53. Lastly, we validated the stability of the binding interaction between the four small molecules and the TP53 through molecular dynamics simulations. The molecular dynamics simulation further validated the strongest binding between TP53 and kaempferol. In essence, our research groundbreaking in its nature elucidates for the first time the underlying molecular mechanism of CQD in the therapeutic management of schistosomiasis, thereby providing valuable insights and guidance for the treatment of this disease.

**Conclusion:**

This study uncovered the efficacious components and underlying molecular mechanisms of the Chufeng Qingpi Decoction in the management of schistosomiasis, thereby offering valuable insights for future fundamental research endeavors.

## 1 Introduction

Schistosomiasis, a prevalent tropical parasitic infection, is predominantly attributed to the blood-dwelling trematodes of the Schistosoma genus. In terms of socioeconomic burden, it ranks prominently as the second among human parasitic diseases (Mawa et al., 2021). For years, blood-dwelling trematodes reside within human blood vessels, deftly evading the immune system while excreting hundreds to thousands of eggs daily. These eggs trigger a distinct immune-mediated granulomatous response, ultimately resulting in a range of local and systemic pathological effects, including anemia, growth retardation, cognitive impairment, and diminished physical fitness (Colley et al., 2014). In the absence of timely intervention, schistosomiasis in its initial stages can progress to advanced forms, posing significant health risks including, but not limited to, upper gastrointestinal hemorrhage, hepatic encephalopathy stemming from infections with Schistosoma japonicum or Schistosoma mansoni, bladder squamous cell carcinoma attributed to Schistosoma haematobium, and ultimately, impairment of fertility (Meilian and Beixing, 2020). Over 250 million individuals in 78 countries across the globe are afflicted with this disease, resulting in approximately 280,000 fatalities each year (LoVerde, 2019).

Currently, schistosomiasis is predominantly managed with chemical drugs in clinical settings. While Praziquantel remains the most effective drug against the disease, its limited efficacy against immature parasites poses a significant challenge. Furthermore, concerns over the emergence of drug resistance loom large due to its widespread and repetitive use in treating large patient cohorts. A vast array of compounds has been evaluated as potential alternatives, but thus far, no suitable substitute or adjuvant has been discovered to supplant Praziquantel (Cioli et al., 2014). The pursuit of novel therapeutic techniques and medications is of utmost importance in the prevention and cure of schistosomiasis. Traditional Chinese medicine, renowned for its distinctive curative effects and minimal side effects, has garnered significant attention in this pursuit.

The traditional Chinese medicine compound prescription Chufeng Qingpi Decoction (CQD) possesses diverse therapeutic attributes, proficiently purifying the spleen, mitigating heat, and concurrently eliminating wind and dampness. This formula is made up of 12 traditional Chinese ingredients, including dried tangerine peel, Forsythia suspensa, Saposhnikovia divaricata, Rhizoma Anemarrhenae, anhydrous sodium sulfate, Scutellaria baicalensis, Scrophularia ningpoensis, Coptidis Rhizoma, schizonepeta spike, rhubarb, Platycodon grandiflorus, and Unprocessed Rehmannia Root. Clinically, it is often used for the treatment of blepharitis, viral blepharitis, schistosomiasis, and follicular keratoconjunctivitis, among other conditions (Xie Lv, 2005; Juying and Jiayi, 2019).

Network pharmacology offers a systematic approach to analyzing the intricate interactions between drugs and diseases, thereby pioneering a novel method to unravel the therapeutic mechanisms underlying traditional Chinese medicine formulas (Peiliang and Hui, 2020). The metabolism of traditional Chinese medicine components, owing to their intricate nature and vast array of therapeutic targets and signaling pathways, exhibits substantial variations among diverse populations. This heterogeneity in metabolic patterns underscores the importance of considering individual differences in clinical applications. This complexity poses a challenge for traditional pharmacology in comprehensively assessing their effects. However, network pharmacologyelucidates the regulatory principles of small molecules in a high-throughput manner, demonstrating remarkable advantages in the analysis of complex systems (Peipei et al., 2012). Consequently, it provides a more profound and scientific rationale for exploring the intricacies of traditional Chinese medicine, offering a robust framework for addressing pertinent research questions. As high-throughput technologies continue to progress, researchers are now empowered to generate copious amounts of high-dimensional data leveraging gene chips, transcriptome sequencing, and related methodologies. Machine learning can be employed to pinpoint disease-related genes, signaling pathways, and functional proteins with precision, so as to identify new drug targets and predict drug efficacy. This streamlined approach significantly accelerates the development of novel therapeutic agents (Cho and Hu, 2022). Molecular docking is an established in silico structure-based method widely used in drug discovery (Pinzi and Rastelli, 2019). In contemporary scientific endeavors, molecular dynamics simulations offer an insightful three-dimensional perspective into molecular binding mechanisms, effectively serving as a valuable adjunct, potential substitute, or even a guiding light for experimental endeavors (Qingran et al., 2017). The integration of these fore fields is not only advantageous for enhancing the efficiency of drug screening but also crucial for enhancing our comprehension of drug action mechanisms.

**Figure 1:**
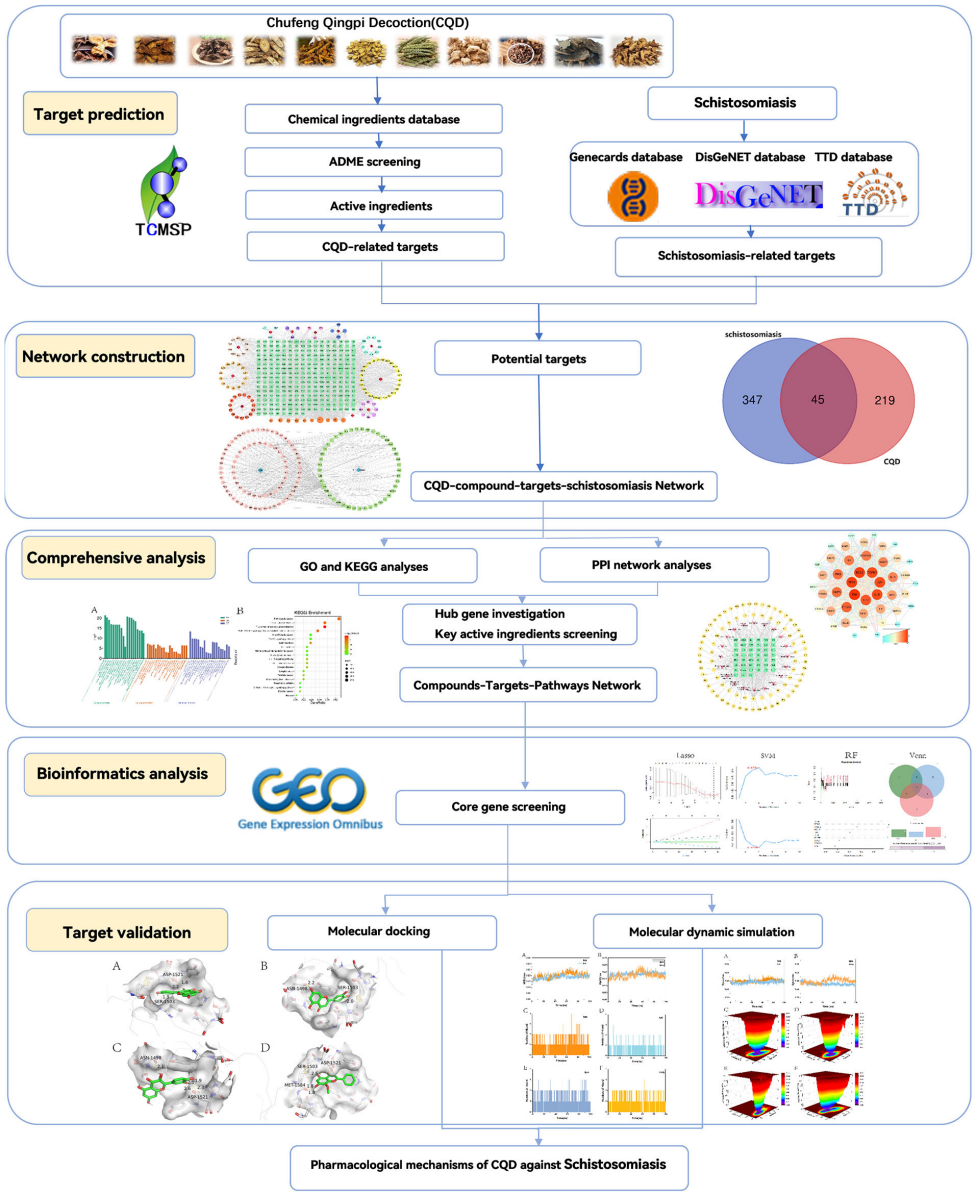
A flow-chart of this study.

Utilizing the network pharmacology methodology, the objective of this study was to pinpoint the pivotal active ingredients within the Chufeng Qingpi Decoction and explore their potential associations with schistosomiasis. Subsequently, we constructed a comprehensive network encompassing the decoction’s components and targets, along with a Protein-Protein Interaction (PPI) network that captures the intricate interplay between chemical and disease-associated targets. To gain insights into the biological mechanisms underlying the therapeutic effectiveness of the decoction, we conducted an in-depth enrichment analysis of potential biological functions and signaling pathways. Furthermore, we constructed a Compounds-Disease Targets Network and a Compounds-Targets-Pathways Network to visualize the complex interplay between compounds, targets, and pathways. To identify the most crucial genes, we subsequently employed three distinct machine learning algorithms: LASSO (Least Absolute Shrinkage and Selection Operator), RF (Random Forest), and SVM-RFE (Support Vector Machine with Recursive Feature Elimination). These algorithms were utilized to sieve through the Protein-Protein Interaction network, prioritizing genes with higher significance scores. Finally, molecular docking and molecular dynamic simulation techniques were employed to simulate drug-target protein interactions, thus validating the accuracy of the network pharmacology predictions. The main scheme of this study is presented in (**Figure 1**).

## 2 Materials and methods

### 2.1 Screening the active ingredients and targets of CQD

The database of Traditional Chinese Medicine Systems Pharmacology (TCMSP) (http://tcmspw.com/tcmsp.php) was utilized to search for the active ingredients and their respective targets of dried tangerine peel, Forsythia suspensa, Saposhnikovia divaricata, Rhizoma Anemarrhenae, Scutellaria baicalensis, Scrophularia ningpoensis, Coptidis Rhizoma, schizonepeta spike, rhubarb, Platycodon grandiflorus, and Unprocessed Rehmannia Root. Sodium sulfate belongs to mineral-based drugs, and several databases such as Genecards, DisGeNET, and OMIM do not contain information on sodium sulfate. Due to its anhydrous nature, sodium sulfate has been excluded from our study. Oral bioavailability (OB) pertains to the fraction of a medication that is absorbed into the systemic circulation after being administered orally. Conversely, drug likeness (DL) serves as a measure of how closely a chemical compound resembles a known pharmacological agent. Prior to further analysis, filtering criteria were established, requiring OB to be at least 30% and DL to reach a minimum of 0.18 (Xiongyang et al., 2008; Chunyan et al., 2024). Subsequently, the principal components and their corresponding target proteins of CQD were identified. Utilizing the Uniprot (https://www.uniprot.org) database, the gene names associated with these protein targets were successfully retrieved.

### 2.2 Construction of active compound-target network

Cytoscape is an effective tool for visualizing network pharmacology and analyzing results (Shannon et al., 2003; Otasek et al., 2019). Utilizing Cytoscape 3.10.0, we imported the efficacious ingredients of CQD and their respective targets, thereby successfully establishing a component-target network for this herbal decoction. The composite target network is composed of nodes that symbolize either a component or a target, with the connections between them being depicted by the lines.

### 2.3 Identification of predicted targets of schistosomiasis

The disease targets of schistosomiasis were searched in the Genecards (https://www.genecards.org/) databases (Stelzer et al., 2016), DisGeNET (DisGeNET - a database of gene-disease associations) databases (Piñero et al., 2020), and OMIM (https://www.omim.org/) databases (Amberger and Hamosh, 2017). The search term “schistosomiasis” was input, and relevant disease-related targets were retrieved from individual databases. Targets procured from both Genecards and DisGeNET were subsequently filtered using a relevance threshold of 0.3 or above. Subsequently, the obtained targets from all three databases were merged, and duplicate values were eliminated. This process culminated in the identification of the disease targets for further investigation.

### 2.4 Construction of PPI network

Simultaneously, we imported the target data of CQD’s active components and those of schistosomiasis into the Draw Venn Diagram program (http://bioinformatics.psb.ugent.be/webtools/Venn/). A Venn diagram was drawn to identify the intersection between the compound targets and the disease targets. The overlapping targets, which are concurrently implicated in both the pathological processes of diseases and the pharmacological mechanisms of drugs, can be preliminarily designated as the therapeutically efficacious targets, whereupon drugs exert their genuine therapeutic effects on diseases. We formulated a comprehensive compounds-disease targets network in order to elucidate the intricate relationships between drug components and their corresponding disease targets. Subsequently, the intersection targets were uploaded to the STRING database (https://string-db.org/) to retrieve protein interaction network information (Szklarczyk et al., 2017). Then, the protein-protein interaction (PPI) data was imported into Cytoscape 3.10.0 to visually represent the intricate network of interactions. Leveraging the CytoNCA plugin within the software, we computed various metrics pertaining to each node in the network diagram, such as Degree, Betweenness Centrality (BC), and Closeness Centrality (CC). These parameters allowed for a comprehensive analysis of the properties of nodes within the interaction network. Betweenness Centrality, a cornerstone metric in the realm of network analysis, quantifies the degree to which a node serves as a pivotal conduit for the shortest paths traversing the network’s fabric. A node exhibiting an elevated Betweenness Centrality underscores its strategic positioning on ital pathways that efficiently interconnect diverse node pairs via the most efficient paths, thereby facilitating the regulation of communication flows and the dissemination of information among the network’s constituents. Conversely, Closeness Centrality, defined as the inverse of the mean geodesic distance from a node to all others within the network, assesses a node’s proximity to the network’s core. A node with a lower average distance to all other nodes attains a superior Closeness Centrality score, indicative of its enhanced capability to rapid interactions and exerting influence over the behavioral patterns of other nodes within the network. Consequently, nodes whose centrality scores surpassed predetermined threshold levels are identified as potential pivotal nodes, crucial for understanding and manipulating network dynamics. Based on the Degree, we predicted 10 potential core goals, selecting those with BC and CC values exceeding their respective median thresholds (Degree > 22.8, BC > 0.012, CC > 0.68) (Duan et al., 2023). Having made these predictions, we proceeded to the next stage of our research.

### 2.5 GO enrichment and KEGG pathway analysis

To investigate the biochemical function of potential schistosomiasis targets, we retrieved Gene Ontology (GO) analysis and Kyoto Encyclopedia of Genes and Genomes (KEGG) data from the Metascape database. The GO analysis serves as a robust tool for delving into biological process (BP), cellular component (CC), and molecular function (MF), thereby enhancing our understanding of their intricacies (Carbon et al., 2017). Moreover, by leveraging KEGG enrichment analysis, we can uncover critical signaling pathways that play a fundamental role in diverse biological processes (Kanehisa et al., 2017). Afterward, the top 20 data points filtered through GO and KEGG were uploaded to the Weishengxin platform (https://www.bioinformatics.com.cn) for visualization (Tang et al., 2023). Furthermore, to visualize the interaction results of components and the outcomes of pathways, we utilized Cytoscape 3.10.0 to create a Compounds-Targets-Pathways Network.

### 2.6 Machine learning

To further optimize the gene selection process, we retrieved gene samples that had been initially screened within the PPI network, specifically those present in the GSE61376 dataset of the GEO database. Following this, we conducted a comprehensive Machine learning by employing cutting-edge algorithms, including LASSO, Random Forest, and Support Vector Machine Recursive Feature Elimination. The Least Absolute Shrinkage and Selection Operator (LASSO) is a technique which simultaneously carries out variable selection and complexity regularization when fitting generalized linear models. This methodology is predominantly employed in the context of protein variable selection, aiming to identify crucial proteins for subsequent modeling and analytical endeavors (Li et al., 2023). A rigorous tenfold cross-validation procedure, utilizing the “glmnet” package, was implemented to precisely differentiate between Schistosomiasis and control samples. Support Vector Machine with Recursive Feature Elimination (SVM-RFE), a sophisticated machine learning approach, was then employed to train a select subset of features from diverse categories, aiming to refine the feature set and identify the most predictive attributes (Zhao et al., 2022). To identify high-quality genes, an SVM-RFE algorithm analysis was conducted utilizing the “e1071” and “svmRadial” packages within the R statistical software framework. Utilizing Random Forest, we prioritized the genes, considering a relative value greater than 0.25 as a significant probabilistic factor (Ishwaran and Kogalur, 2010). An RF classification model was established utilizing the “Random Forest” software package to prioritize key genes based on the Gini index, thereby screening for targets of characteristic expression. Upon convergence of the three machine learning algorithms, the genes that intersect are identified as crucial components for the treatment of Schistosomiasis with CQD.

### 2.7 Molecular docking

We accessed the PubChem database (https://pubchem.ncbi.nlm.nih.gov/) to obtain the three-dimensional structure of the key chemical components of CQD (Wang et al., 2012). This structure was then converted into PDB format using PyMOL. Next, we utilized AutoDockTools to modify the composition structure, calculate the charge, determine the distortion degree, and designated it as the ligand. Subsequently, based on high-resolution data for human species, we retrieved protein structures associated with disease targets from the RCSB (https://www.rcsb.org/) database. Following the removal of free water molecules, the addition of hydrogen atoms, and the computation of charges, the proteins were designated as macromolecules. Molecular docking was then conducted on both ligands and macromolecules, with the establishment of suitable docking boxes tailored to the protein size, ultimately leading to the generation of “DLG” files. Finally, we selected the 3D conformation with the optimal affinity and most stable intermolecular forces, exported it as a “PDBQT” file, and visualized it through PyMOL (Wu et al., 2022).

### 2.8 Molecular dynamic simulation

We conducted molecular dynamics simulations to gain an in-depth comprehension of the receptor-ligand complex’s interaction strength and stability. We utilized the Gromacs 2024 software for these dynamic simulations. Before initiating the simulations, the topology file for small molecules was generated via the sobtop tool, while the protein’s molecular structure and corresponding topology file were prepared using the built-in commands in Gromacs. To simulate the physiological environment accurately, we employed the Amber14sb force field and SPC model water molecules (Ponder and Case, 2003), maintaining a minimum buffer distance of 20 Å between protein atoms and the water box’s edge. System neutrality was achieved by setting the salt concentration at 0.15 mol/L. Before the actual simulation, the system’s molecular mechanics were optimized using a maximum steep descent method with 50,000 steps. Subsequently, the optimized system underwent NVT and NPT ensemble equilibration, employing a step size of 2 fs and a total duration of 100 ps, while constraining the system’s position during this process. Following equilibration, a 100-ns MD simulation was performed at 310K with a 2fs time interval. Using GROMACS’s built-in tools, the trajectory was extracted with the final structure at 100 ns, having eliminated periodic boundary conditions (Hess et al., 2008; Pronk et al., 2013). This structure was then superimposed onto the initial complex structure to analyze the interactions between the protein and small molecule. Subsequently, we evaluated key metrics including the root mean square deviation (RMSD) and protein gyration radius of the protein-small molecule complex. Gibbs free energy landscape was drawn. The binding free energy between the receptor and ligand was calculated using the gmx_MMPBSA method (Valdés-Tresanco et al., 2021). Finally, visualization of the simulation results was achieved using Pymol 4.6.0 and Origin 2024 software.

## 3 Results

### 3.1 Compound-target network and analysis

**Figure 2:**
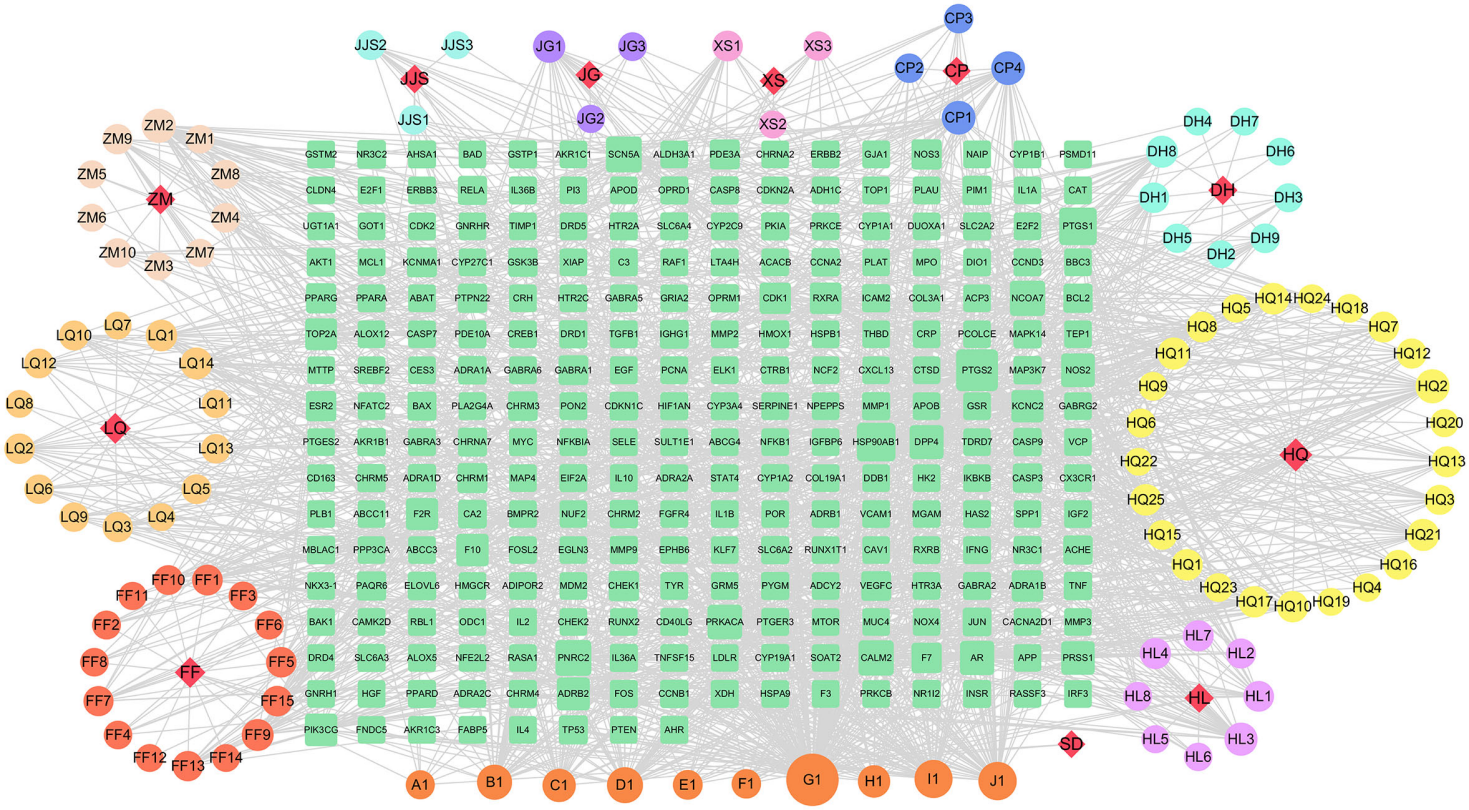
Herbal medicine-compound-target network of CQD. (11 red diamonds represent the main drug components of CQD, the circles around the diamonds represent the main components of each drug, the bottom 10 orange circles represent common components, and the 264 green squares in the middle represent drug targets).

Based on the distinctive characteristics of the diverse components and targeted receptors within traditional Chinese medicine formulas, we have devised a comprehensive component-target interaction network specific to CQD. As depicted in (**Figure 2**), a total of 379 nodes and 1629 edges were identified, with green squares representing potential targets for wind removal and spleen clearing. We retrieved effective ingredients and their corresponding targets from the TCMSP database for Forsythia suspensa, Saposhnikovia divaricata, Rhizoma Anemarrhenae, Scutellaria baicalensis, Scrophularia ningpoensis, Coptidis Rhizoma, schizonepeta spike, rhubarb, Platycodon grandiflorus, and Unprocessed Rehmannia Root, specifically focusing on the 11 components of the Chufeng Qingpi Decoction. Sodium sulfate, an inorganic compound formed by the weathering of mirabilite and the loss of crystalline water, was excluded from the analysis. Additionally, we substituted dried tangerine peel for its intended component. The conversion of each protein target into its corresponding gene names was achieved through the utilization of the Uniprot database. Given the extensive MOLIDs associated with each component in the compound, they were numbered, and their respective relationships were clearly presented in (**Supplementary Table 1**). Following a thorough analysis of the calculated metrics, namely Degree, BC, and CC, we successfully identified the four most important components: wogonin, kaempferol, luteolin, and quercetin, which proved to be the primary active ingredients. The OB values, DL values, and molecular structures of these four components were shown in (**Table 1**).

**Table 1:** Top four compounds information of CQD network.

MOL ID	Compound	Molecule Structure	Degree	OB (%)	DL
MOL000006	luteolin	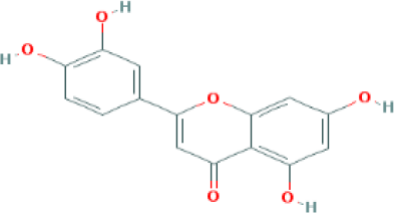	60	36.16	0.25
MOL000422	kaempferol	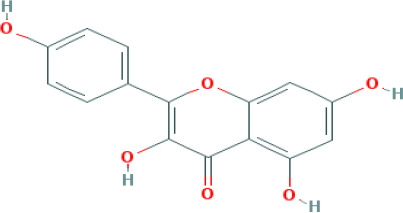	61	41.88	0.24
MOL000098	quercetin	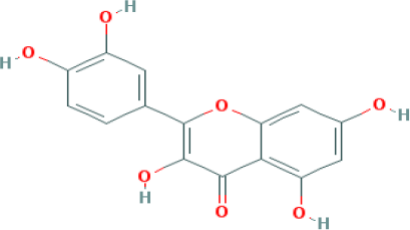	140	46.43	0.28
MOL000173	wogonin	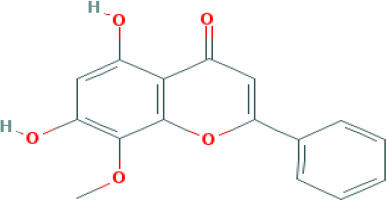	48	30.68	0.23

### 3.2 PPI network construction and analysis

**Figure 3:**
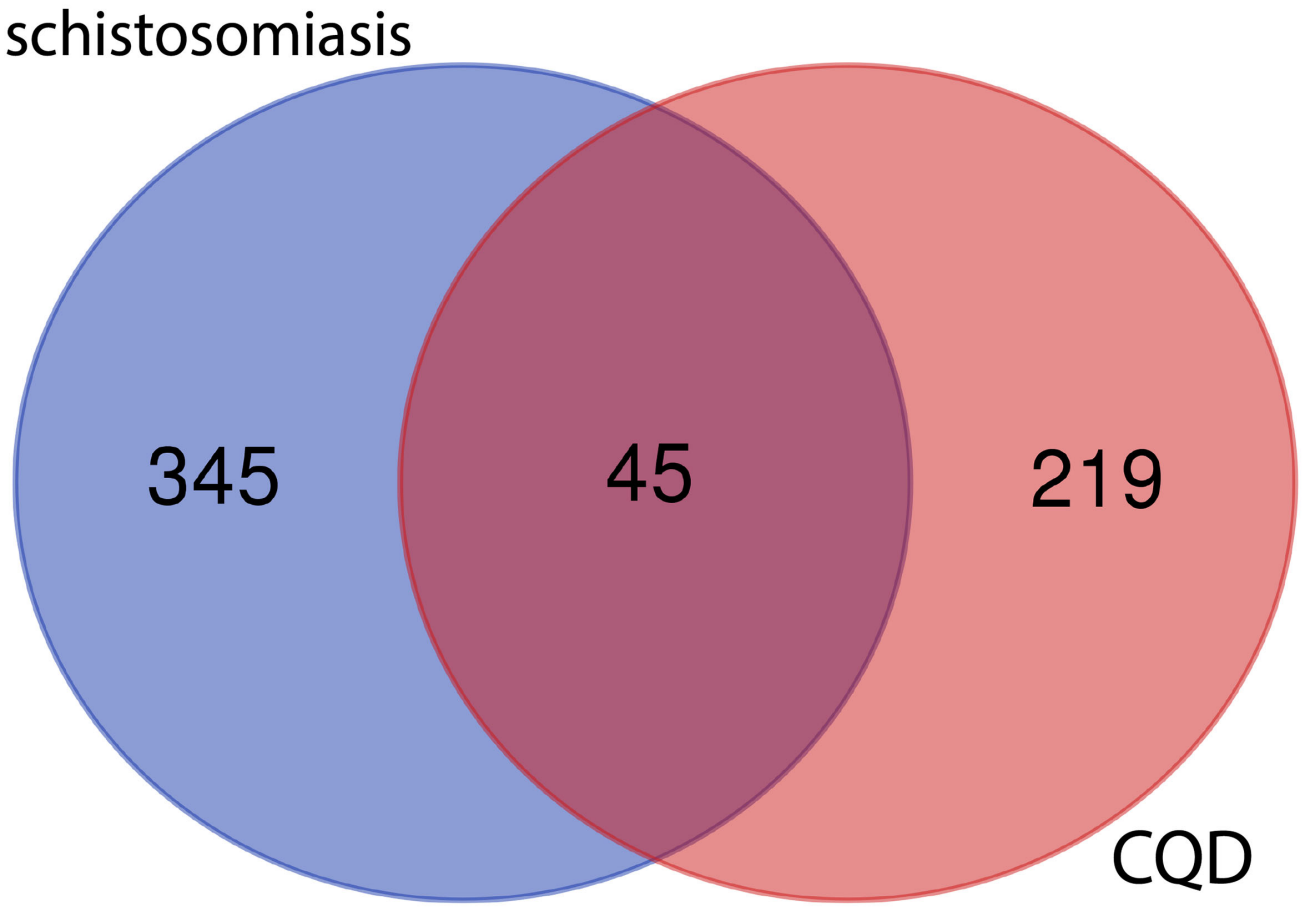
Venn diagram of the target of CQD and the target of sepsis.

**Figure 4:**
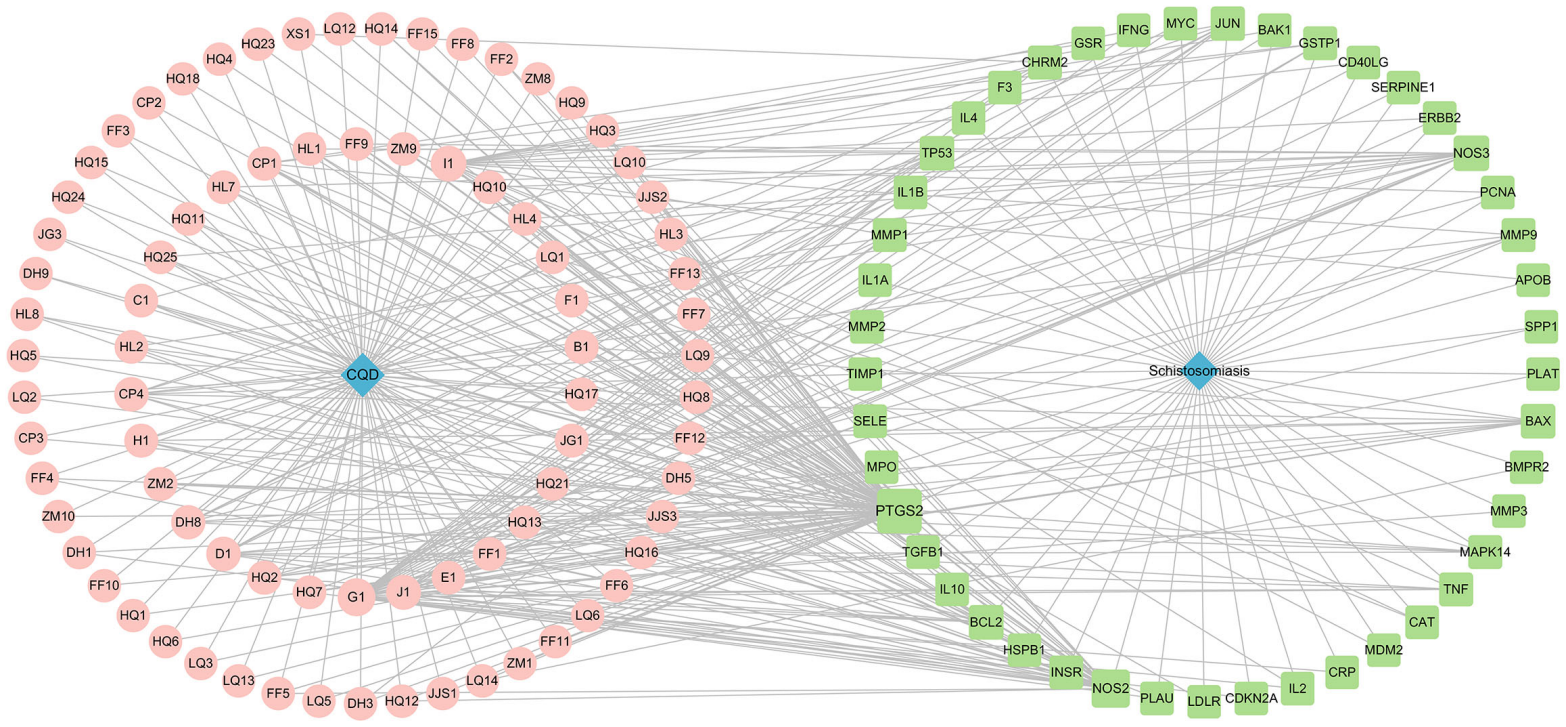
Compounds-Disease targets network (Pink represents drug ingredients, green represents disease targets).

**Figure 5:**
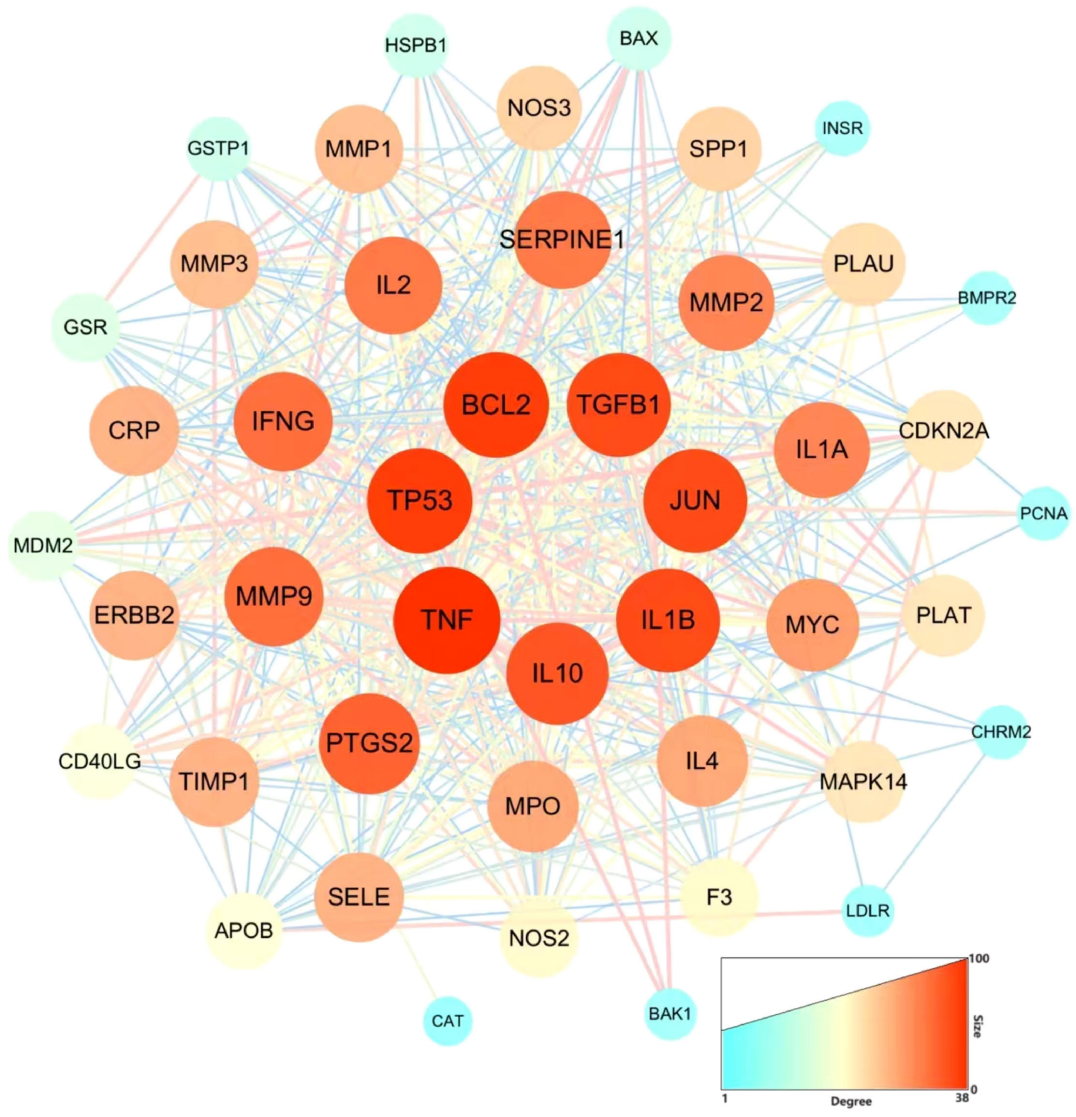
The PPI network of CQD and sepsis targets. Nodes represent proteins (The colors from blue to red represent the degree of binding between proteins). Edge represents protein-protein association.

After screening the active targets of schistosomiasis through the disease database and eliminating duplicate values, we identified 390 disease targets. We then utilized these data to generate a Venn diagram (**Figure 3**), comparing the active target components of the disease with those present in the compound formulas. The intersection of these sets exposed 45 shared objectives between the active elements of CQD and schistosomiasis. To elucidate the intricate relationships between drug components and their corresponding disease targets, we formulated a comprehensive compounds-disease targets network (**Figure 4**). To illustrate these shared targets, we crafted a PPI network encompassing 45 nodes interconnected by 513 edges (**Figure 5**). In this network, the size and opacity of a node corresponded to its degree value, indicating the likelihood of it being a core target. Finally, we narrowed down our focus to the top 10 targets by applying threshold values for Degree >22.8, BC >0.012, and CC >0.68 (**Table 2**).

**Table 2:** Top 10 targets information of PPI network.

Name	BetweennessCentrality	ClosenessCentrality	Degree
TNF	0.036966652	0.88	38
TP53	0.068828962	0.862745098	37
BCL2	0.058120684	0.862745098	37
IL1B	0.036197714	0.846153846	36
TGFB1	0.034777133	0.830188679	36
JUN	0.022347974	0.846153846	36
IL10	0.031105562	0.830188679	35
PTGS2	0.017361014	0.814814815	34
MMP2	0.042925395	0.75862069	31
MYC	0.020191104	0.733333333	29

### 3.3 GO enrichment and KEGG pathway analysis

**Figure 6:**
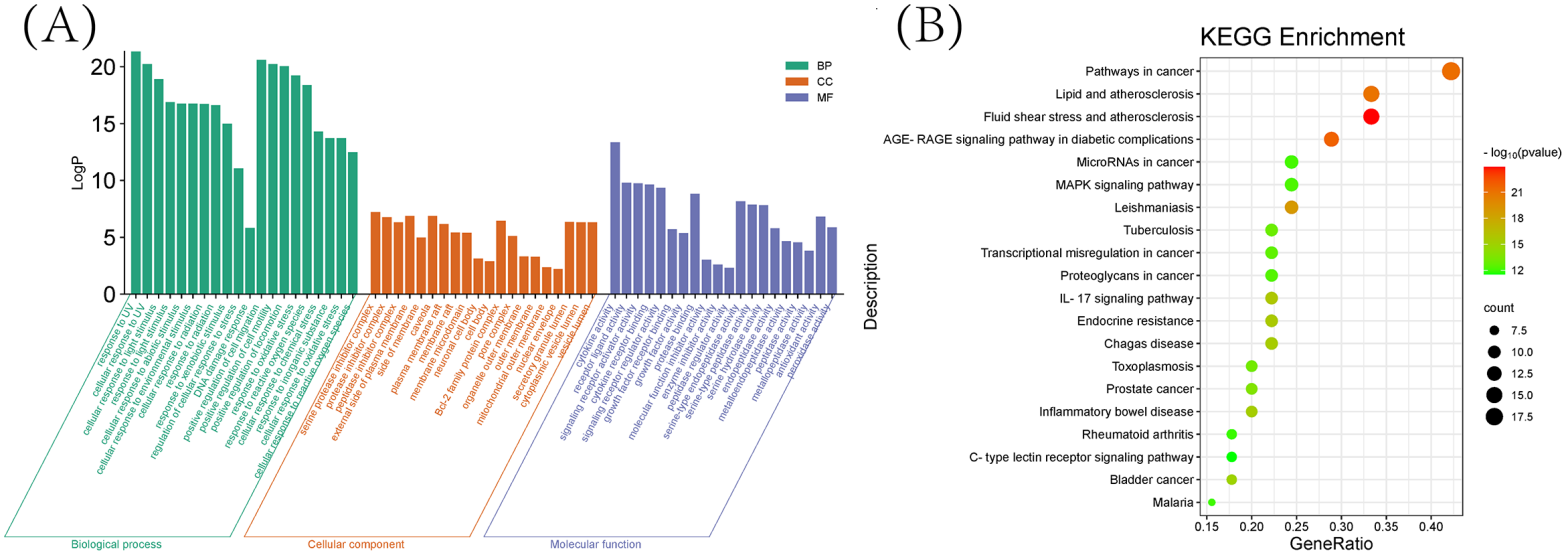
Enrichment analysis **(A)** Top 20 GO terms of hub genes. **(B)** Top 20 KEGG pathway of hub genes.

**Figure 7:**
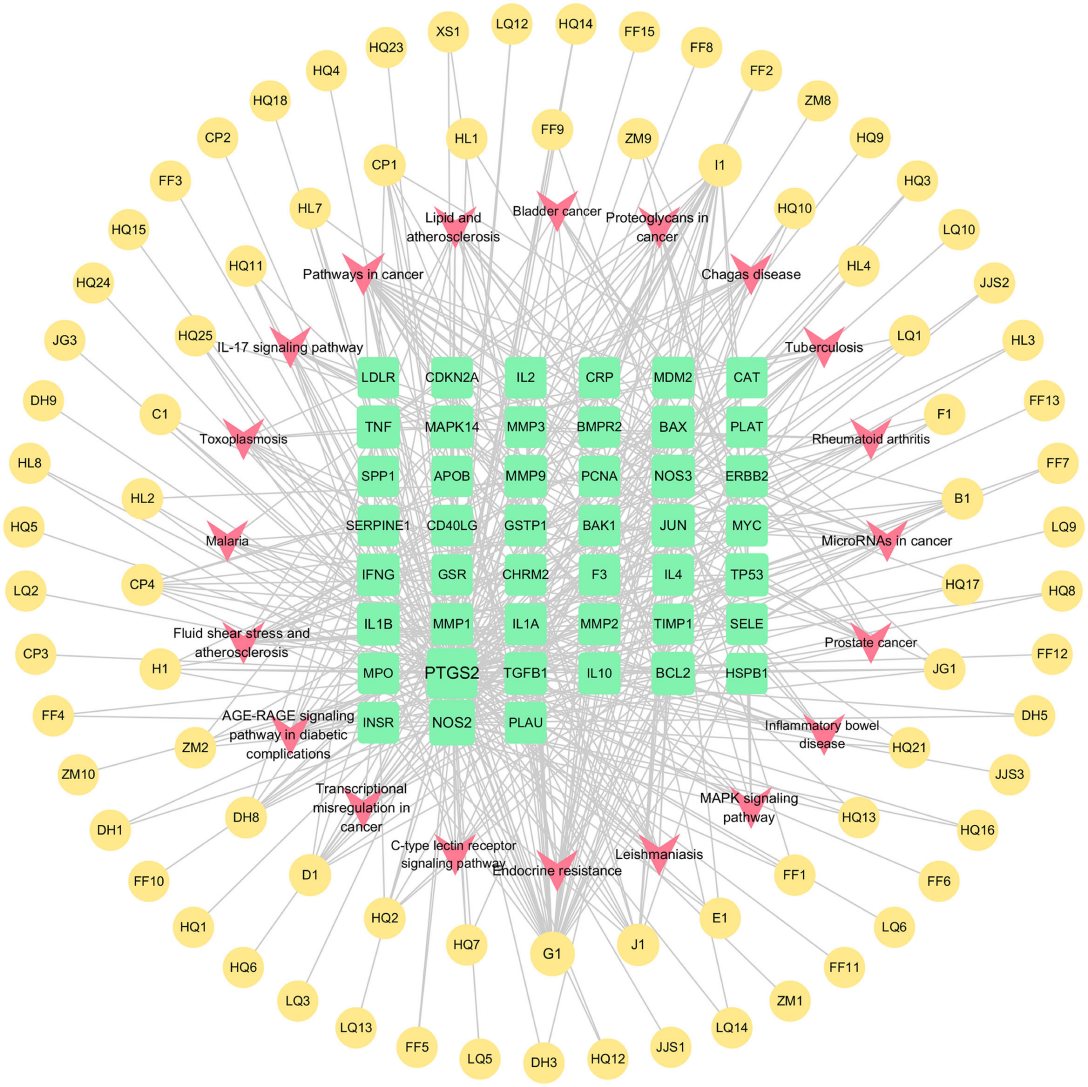
Compounds-Targets-Pathways Network (The yellow circle around represents the active ingredients of CQD, the green square in the center represents the screened disease targets, and the pink part represents the KEGG pathway).

As shown in (**Figure 6A**), we previously performed a Gene Ontology (GO) analysis to identify 20 pertinent biological processes, cellular components, and molecular functions, including responses to UV radiation, positive regulation of cell migration and motility, alongside other crucial biological phenomena. Subsequently, to delve into the signaling pathway mechanism underlying the treatment of schistosomiasis with CQD, we performed a KEGG enrichment analysis. The resulting (**Figure 6B**) highlights the top 20 signaling pathways, which include Pathways in cancer, Lipid and atherosclerosis, Fluid shear stress and atherosclerosis, as well as the AGE-RAGE signaling pathway. Furthermore, to visualize the interaction results of components and the outcomes of pathways, we utilized Cytoscape 3.10.0 to create a Compounds-Targets-Pathways Network (**Figure 7**). It describes the interactions between drugs and their targets as well as the locations and functions of these targets in the signaling pathways within the organism.

### 3.4 Machine learning

**Figure 8:**
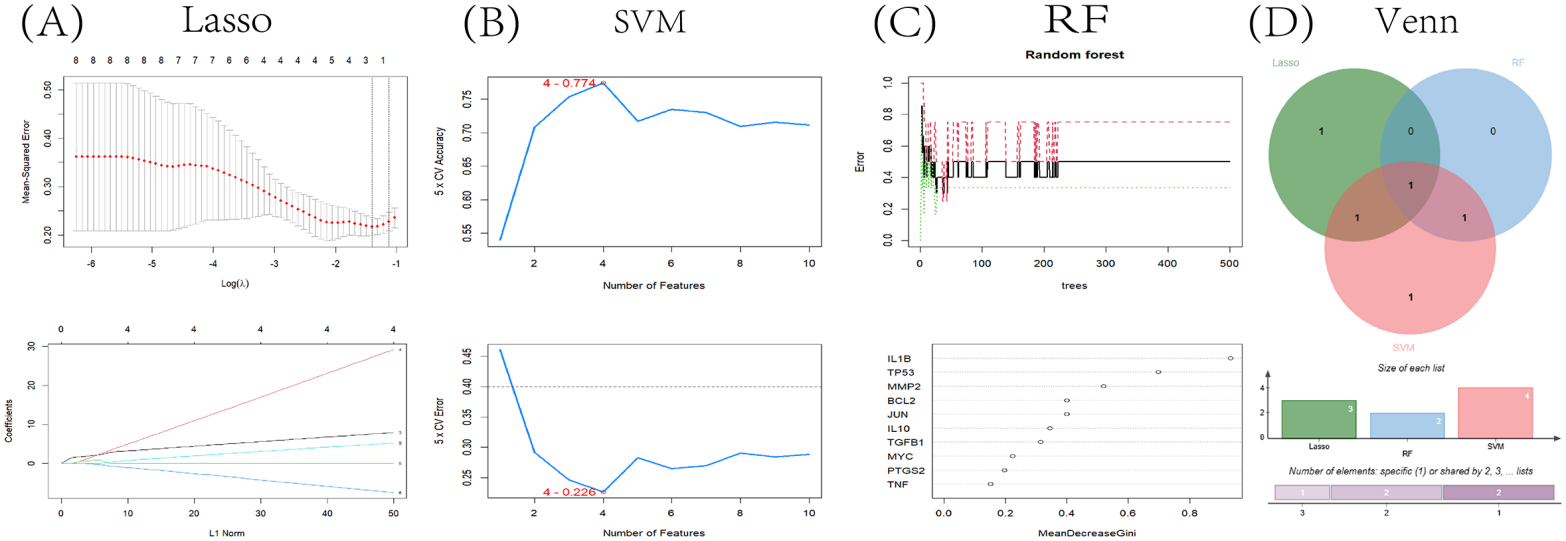
Bioinformatic analysis. **(A)** Using the LASSO logistic regression algorithm with tenfold cross-validation to adjust penalized parameter and select three related genes; **(B)** SVM-RFE algorithm to filter 10 key genes to determine the best combination of key genes. Finally, 4 genes (maximum precision=0.774, minimum RMSE=0.226) were identified as the best key gene; **(C)** Key gene screening was performed by random forest algorithm, and 2 genes were identified as key genes based on gene importance greater than 0.6; **(D)** Identifying key gene TP53 through Venn diagram as intersecting targets.

Utilizing Machine learning, we conclusively identified the core gene, TP53, by screening for significant key genes in the training dataset via three distinct machine learning algorithms: LASSO, SVM-RFE, and RF. The LASSO algorithm narrowed down the initial 10 genes to three crucial ones (**Figure 8A**). In parallel, the SVM-RFE algorithm yielded four genes, achieving a peak accuracy of 0.774 and a minimal RMSE of 0.226 (**Figure 8B**). Additionally, the RF algorithm assigned an importance score to each gene, and we established a threshold of 0.6, resulting in the identification of two key genes (**Figure 8C**). Ultimately, the intersection of these three gene sets identified a single core gene, TP53, for subsequent research (**Figure 8D**).

### 3.5 Molecular docking study

**Figure 9:**
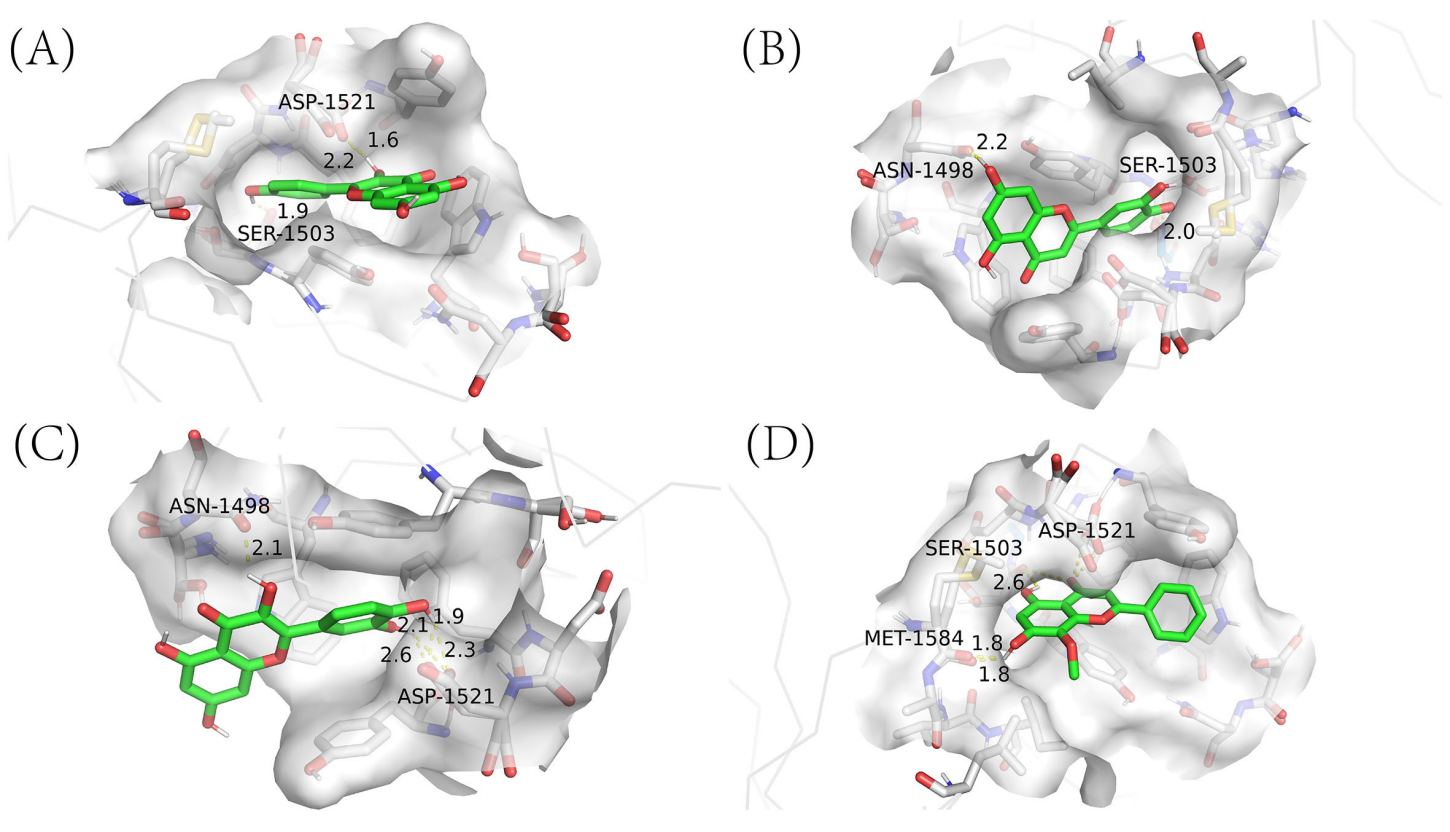
Molecular docking results of main chemical components of CQD. **(A)** kaempferol -TP53; **(B)** luteolin -TP53; **(C)** quercetin -TP53; **(D)** wogonin -TP53.

Utilizing network pharmacology, we have previously pinpointed four essential active ingredients of CQD: wogonin, kaempferol, luteolin, and quercetin. Subsequently, we performed molecular docking analysis of these compounds with 2G3R protein encoded by TP53 (**Figure 9**). This analysis aimed to establish the pivotal role of these compounds in the treatment of schistosomiasis. Subsequently, we scrutinized the docking results of proteins and ligands separately, with the Autodock outcomes being summarized in the (**Table 3**). It is widely recognized that a lower binding energy between molecules suggests a stronger affinity. Typically, binding energies below -5 kj/mol are indicative of robust binding between ligands and receptors (Ezebuo and Uzochukwu, 2022). Our findings demonstrate that all four small molecules possess favorable binding affinities towards the TP53-encoded protein. Notably, kaempferol exhibits the most favorable binding energy, indicative of a particularly robust interaction with TP53. Furthermore, the interactions between all ligand-macromolecule complexes are mediated by hydrogen bonds, thereby ensuring the stability of these bindings.

**Table 3:** Autodock outcomes.

Compounds	Binding energies(kj/mol)
kaempferol -TP53	-7.26
luteolin -TP53	-6.74
quercetin -TP53	-5.6
wogonin -TP53	-7.23

### 3.6 Molecular dynamic simulation

**Figure 10:**
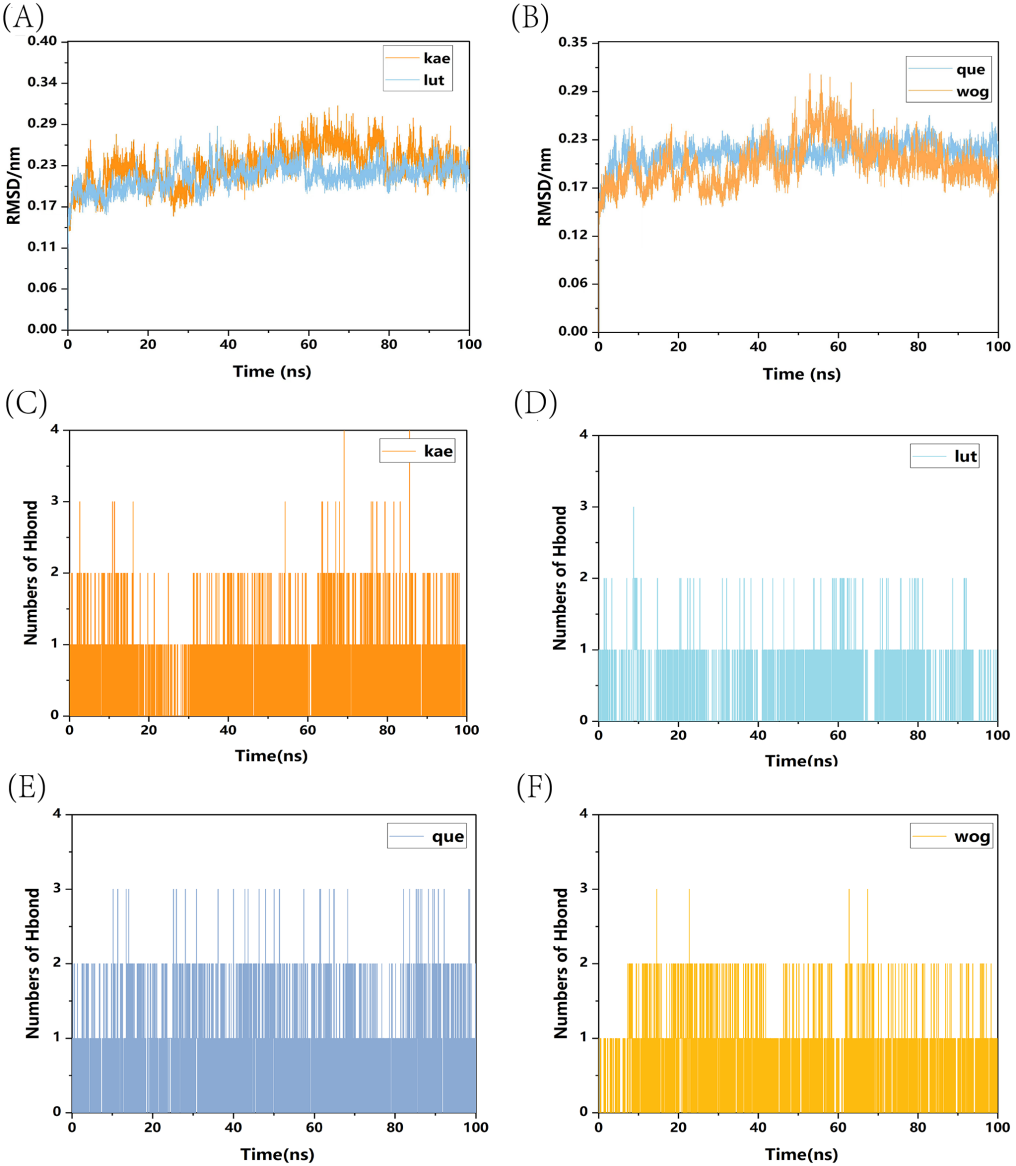
RMSD curves and hydrogen bond numbers of four complexes. **(A)** RMSD curves of TP53-kaempferol and TP53-luteolin; **(B)** RMSD curves of TP53-quercetin and TP53-wogonin; **(C)** The total number of hydrogen bonds in TP53-kaempferol; **(D)** The total number of hydrogen bonds in TP53-luteolin; **(E)** The total number of hydrogen bonds in TP53-quercetin; **(F)** The total number of hydrogen bonds in TP53-wogonin.

**Figure 11:**
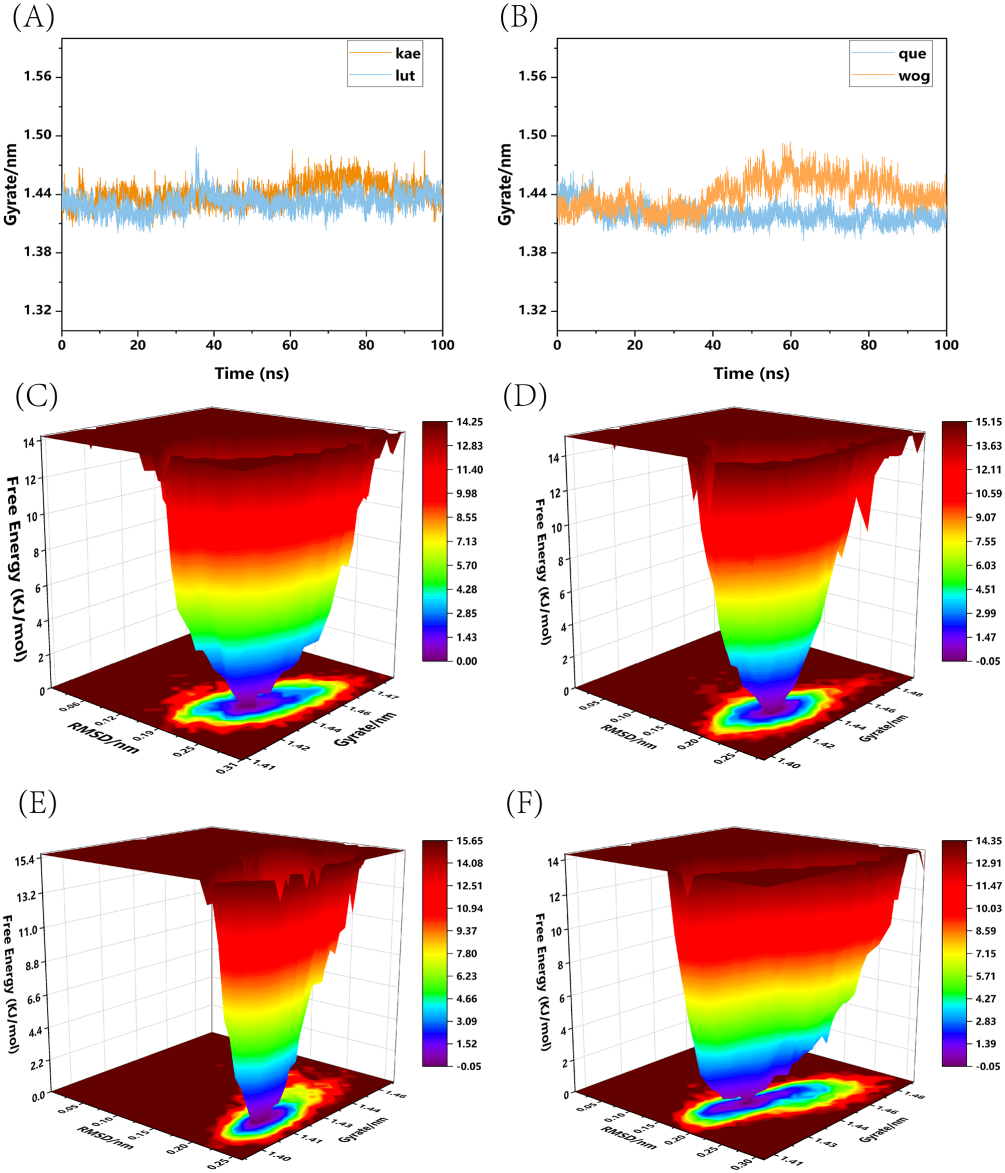
Rg curves and Gibbs free energy landscape maps of four types of complexes. **(A)** Rg curves of TP53-kaempferol and TP53-luteolin; **(B)** Rg curves of TP53-quercetin and TP53-wogonin; **(C)** Gibbs free energy landscape map of TP53-kaempferol; **(D)** Gibbs free energy landscape map of TP53-luteolin; **(E)** Gibbs free energy landscape map of TP53-quercetin; **(F)** Gibbs free energy landscape map of TP53-wogonin.

We conducted a standard 100-nanosecond molecular dynamics simulation on the ligand-receptor complex system. The RMSD (Root Mean Square Deviation) curve serves as an indicator of protein conformation fluctuations (Brüschweiler, 2003). In (**Figure 10A**), the RMSD values of TP53 bound with kaempferol and luteolin are displayed, both fluctuating between 0.14 and 0.29 nm throughout the simulation; whereas (**Figure 10B**) shows the RMSD of TP53 complexes with quercetin and wogonin, fluctuating between 0.145 and 0.29 nm throughout the simulation. The RMSD analysis encompassed the entire structure of these complexes. The RMSD curves depicted in the figures reveal that all four complexes demonstrate minimal fluctuations during the 0-100ns time frame, signifying their binding stability. Hydrogen bonding stands as a potent non-covalent interaction force. Over the 0-100ns duration, TP53-kaempferol forms 0-4 hydrogen bonds (**Figure 10C**), whereas TP53-luteolin (**Figure 10D**), TP53-quercetin (**Figure 10E**), and TP53-wogonin (**Figure 10F**) maintain a hydrogen bond count within the range of 0-3. Furthermore, following molecular dynamics simulations, hydrogen bonds, the binding of TP53 to the four small molecule substances also involves other chemical bonds. We have illustrated the planar graph after the second docking in (**Supplementary Figure 1**), which provides a more detailed view of the interactions between the two. These hydrogen bonds play a crucial role in preserving the ligand-receptor binding stability. The radius of gyration (Rg) serves as an indicator of both the compactness of molecular constraints and the overall system’s constraint level, while also reflecting the extent of protein folding (Lobanov et al., 2008). A smaller Rg value signifies a system that is densely and tightly packed. As illustrated in the accompanying figure, the four complexes exhibit stable fluctuations within the 0-100ns timeframe. Specifically, the Rg values for TP53-kaempferol and TP53-luteolin remain steady within the 1.41 nm to 1.47 nm range (**Figure 11A**), TP53-quercetin stabilizes within 1.41 nm to 1.44 nm, and TP53-wogonin oscillates between 1.41 nm and 1.48 nm (**Figure 11B**). Gibbs free energy landscapes reveal intricate stability patterns (Bharatiy et al., 2016). To identify and investigate the stable conformations, we employed RMSD and Gyrate metrics to generate the landscape visualizations. Within these landscapes, the blue and purple zones signify that the stable conformations of the complexes are represented in regions of minimal free energy at lower energetic states (Ali et al., 2019). Specifically, the TP53-kaempferol complex attains a notably stable conformation when the Rg value falls between 1.42 nm and 1.44 nm, coupled with an RMSD value ranging from 0.22 nm to 0.25 nm (**Figure 11C**). In the case of the TP53-luteolin complex, the free energy reaches a minimum when the Rg value spans from 1.41 nm to 1.44 nm, accompanied by an RMSD value within 0.20 nm to 0.225 nm (**Figure 11D**). Analogously, the TP53-quercetin complex demonstrates a significantly stable conformation with an Rg value between 1.405 nm and 1.42 nm, and an RMSD value in the range of 0.2125 nm to 0.2375 nm (**Figure 11E**). Ultimately, the TP53-wogonin complex attains the lowest free energy state when the Rg value is between 1.42 nm and 1.45 nm, corresponding to an RMSD value of 0.175 nm to 0.2 nm (**Figure 11F**). Following the observed stability in RMSD curves across the entire simulation, we proceeded to compute the binding free energy within the 0-100 ns time window. The overall binding free energy of the compound composed of TP53 and the other four small molecules was shown in (**Supplementary Figure 2**). Our findings reveal that the TP53-kaempferol complex possesses an average binding free energy of -22.80 kcal/mol, accompanied by an average van der Waals energy of -32.17kcal/mol and an electrostatic energy of -8.79 kcal/mol. In the case of the TP53-luteolin complex, the average binding free energy stands at -20.88 kcal/mol, with a corresponding average van der Waals energy of -27.70 kcal/mol and an electrostatic energy of -7.74 kcal/mol. The TP53-quercetin complex showcases an average binding free energy of -19.17 kcal/mol, coupled with an average van der Waals energy of -27.62 kcal/mol and a notable electrostatic energy of -185.21 kcal/mol. Finally, the TP53-wogonin complex demonstrates an average binding free energy of -21.15 kcal/mol, an average van der Waals energy of -29.67 kcal/mol, and an electrostatic energy of -124.10 kcal/mol. The aforementioned findings demonstrate the existence of stable interactions between the four small molecules and the expressed TP53 proteins, subsequently bolstering the reliability of molecular docking predictions.

## 4 Discussion

Our research sought to explore the possible therapeutic effects of the Chufeng Qingpi Decoction in treating schistosomiasis. Utilizing the TCMSP database, 104 chemical components were identified, with wogonin, kaempferol, luteolin, and quercetin being the most prominent. These primary components were found to interact with various other active ingredients, targeting multiple networks. Specifically, wogonin, kaempferol, and quercetin have been empirically proven to exert therapeutic benefits in treating schistosomiasis.

Wogonin, the primary bioactive component of the Chufeng Qingpi Decoction, has been empirically validated in studies to significantly enhance the histopathological condition of the liver in mice infected with Schistosoma mansoni when combined with praziquantel, effectively alleviating the burden of hepatic eggs (Pekkle Lam et al., 2022). Furthermore, its deworming properties contribute to further mitigation of disease progression.

Kaempferol, another active ingredient in the Chufeng Qingpi Decoction, effectively reduces the expression of TGF-β1 and Smad2/3 while enhancing the expression of Smad7 (Cai et al., 2014), thereby significantly mitigating the severity of liver fibrosis and granuloma caused by schistosome eggs subsequent to treatment with praziquantel.

Quercetin displays a notable capacity to reduce liver fibrosis, evident through its substantial downregulation of early response genes such as TIMP-1 (tissue inhibitor of metalloproteinase 1), as well as type I and III collagen (Xu et al., 2006). Furthermore, when compared to the praziquantel treatment group, the quercetin group displayed a notable reduction in the expression of c-jun mRNA, as well as type I and III collagen, suggesting that quercetin may potentially exert a superior long-term effect on schistosomiasis-induced liver fibrosis compared to praziquantel.

In summary, the traditional Chinese medicine formula of the Chufeng Qingpi Decoction, comprising diverse components, holds promise for the effective treatment of schistosomiasis. Its primary constituents—wogonin, kaempferol, luteolin, and quercetin—synergistically target distinct pathological aspects of the disease, thereby achieving therapeutic objectives. These revelations underscore the significance of the Chufeng Qingpi Decoction in schistosomiasis management and justify its further exploration.

By retrieving disease-related targets for schistosomiasis from the Genecards, DisGeNET, and OMIM databases, we identified 45 potential targets that intersected with the compound of interest. This led to the discovery of promising therapeutic targets for the treatment using Chufeng Qingpi Decoction. To gain deeper insights into these core targets, we constructed a PPI network to visually represent the interactions between proteins. Subsequently, we utilized Machine learning algorithms, including LASSO, SVM-RFE, and RF, to predict the most crucial gene, TP53.

TP53, a crucial tumor suppressor gene, is vulnerable to mutations caused by the combined influence of schistosomiasis and aflatoxin B1 exposure, leading to an increased frequency of p53 mutations (Habib et al., 2006). This escalated mutation rate in p53 may expedite the progression of early hepatocellular carcinoma in patients with schistosomiasis. Furthermore, endogenous genotoxic substances produced during Schistosoma mansoni infection disrupt cell apoptosis by modifying the expression pattern of Bcl-2 protein (Zalata et al., 2005).

To delve into the therapeutic mechanism of the Chufeng Qingpi Decoction in treating schistosomiasis, we conducted GO analysis and KEGG enrichment analysis. The GO analysis revealed that the target genes are primarily enriched in processes including UV response, positive regulation of cell migration, and cell motility. These discoveries serve as a theoretical foundation for further elucidating the therapeutic mechanisms of the Chufeng Qingpi Decoction in schistosomiasis treatment.

The results of the KEGG enrichment analysis highlighted the involvement of a diverse range of signaling pathways in the therapeutic effect of the Chufeng Qingpi Decoction against schistosomiasis. Notably, Pathways in cancer, as well as Lipid metabolism, Atherosclerosis, Fluid shear stress and atherosclerosis, and the AGE-RAGE signaling pathway, play significant roles in this process. Notably, the enrichment of Pathways in cancer is particularly significant, suggesting that this pathway could be a key signaling mechanism in the treatment of schistosomiasis. Pathways in cancer refer to signaling cascades crucial to the initiation and development of cancer, encompassing the PI3K/Akt/mTOR, Wnt/β-catenin, and MAPK signaling pathways. Remarkably, the Wnt/β-catenin signaling pathway, a well-established PF pathway, assumes a critical role in regulating cellular proliferation, inflammatory cell infiltration, neovascularization in lung tissue, and the epithelial-mesenchymal transition (EMT) process (Xin et al., 2024). Research has convincingly demonstrated that infection with Schistosoma mansoni, along with its soluble egg antigen (SEA), and eggs of the parasite, are capable of triggering the Wnt/β-catenin signaling pathway and subsequently activating the proto-oncogene c-Jun in intestinal cells (Weglage et al., 2020). These insights offer a refined theoretical framework for understanding the therapeutic mechanisms of the Chufeng Qingpi Decoction in the treatment of schistosomiasis.

To investigate the potential molecular mechanism underlying the traditional medicinal approach of removing wind and clearing spleen in the treatment of schistosomiasis, we capitalized on previous network pharmacology research outcomes, specifically the screening of bioactive ingredients in compound formulas and PPI network target screenings. After rigorous Machine learning of the previously identified key genes, we successfully pinpointed the most crucial gene. Consequently, our studies revealed four pivotal bioactive components: wogonin, kaempferol, luteolin, and quercetin, alongside a representative target. To elucidate the interaction mechanisms, we conducted molecular docking and molecular dynamics simulations of these components with the protein encoded by TP53. Following this, our predictions in network pharmacology underwent validation via molecular docking experiments. The docking analysis revealed that kaempferol exhibited the lowest binding energy upon interacting with TP53, thereby indicating a highly stable complex formation. The molecular dynamics simulation results uncovered stable binding conformations between four small molecules and TP53. In conclusion, our comprehensive research endeavors underscore kaempferol as a pivotal constituent within CQD, exhibiting paramount significance in the therapeutic armamentarium against schistosomiasis. Notably, TP53 emerges as the primary target of kaempferol, highlighting its critical role and fostering promising avenues for intensified future investigations in this domain. Furthermore, the intricate physiological processes and associated signaling pathways that underlie the drug’s mechanism offer invaluable insights and serve as navigational guideposts for subsequent research endeavors aimed at advancing the therapeutic landscape for schistosomiasis.

This study inevitably has certain limitations. First of all, the accuracy and timeliness of database information must be further improved, as the predictive outcomes of network pharmacology are heavily reliant on extensive bioinformatics data. Furthermore, while network pharmacology predominantly focuses on exploring interactions between drugs and biological systems, it fails to fully consider the complex and integrated characteristics inherent in traditional Chinese medicine formulae. This limitation may impede a thorough comprehension of their comprehensive therapeutic mechanisms. Additionally, molecular docking and dynamics simulations are primarily utilized to mimic drug-target engagements, yet traditional Chinese medicine formulations frequently encompass a variety of targets and therapeutic avenues. This complexity poses a challenge to the extent of which these strategies can comprehensively decipher the intricate therapeutic mechanisms underlying these formulations. Furthermore, our research has merely unveiled the crucial targets of CQD in schistosomiasis treatment, touching upon the biological mechanisms and key pathways without conducting further experimental validations of the specific underlying mechanisms. This presents a certain limitation compared to studies that delve into the detailed biochemical reactions occurring within cells after schistosome infection and the effects of decoctions thereon (Luo et al., 2022).Moreover, our research has primarily identified pivotal targets of CQD in schistosomiasis therapy, offering initial glimpses into the biological mechanisms and pivotal pathways, albeit without conducting exhaustive experimental validations to unravel the intricate underlying mechanisms. This constitutes a limitation in comparison to studies that meticulously examine the intricate biochemical cascades within cells subsequent to schistosome infection and the nuanced effects elicited by decoctions. Future endeavors should strive to bridge this gap through comprehensive experimental validations, thereby advancing our understanding of the therapeutic mechanisms of CQD in schistosomiasis.

In this study, wogonin, kaempferol, luteolin, and quercetin have been pinpointed as the four key bioactive components for schistosomiasis treatment using Chufeng Qingpi Decoction. However, these components alone cannot encapsulate the complexity of the Chufeng Qingpi Decoction, and disparities persist between our theoretical model and the intricate *in vivo* setting. Consequently, it is imperative to complement our pharmacological experiments with actual clinical data to solidify our findings. Furthermore, our investigation has revealed that prior studies have been somewhat restrictive in exploring these results. The elusive nature of the effects and underlying mechanisms of these potential bioactive compounds on schistosomiasis necessitates further elucidation and rigorous validation. Consequently, we posit that there exists substantial scope for development and profound research significance in this area.

## 5 Conclusion

In this study, we conducted an in-depth exploration of the key chemical components and underlying mechanisms of the Chufeng Qingpi Decoction in the treatment of schistosomiasis. Utilizing a multi-faceted approach encompassing network pharmacology, machine learning, molecular docking, and molecular dynamics simulations, this comprehensive analysis aimed to gain a thorough understanding of the decoction’s therapeutic capabilities. The results obtained indicate that wogonin, kaempferol, luteolin, and quercetin are likely the principal active components of the Chufeng Qingpi Decoction. Furthermore, these components were found to target proteins such as PTGS2, TNF, TGFB1, BCL2, TP53, IL10, JUN, MMP2, IL1B and MYC, suggesting their potential as therapeutic targets for schistosomiasis treatment. Among the identified targets, TP53 emerges as the pivotal target of utmost significance. Additionally, it was postulated that these components might exert therapeutic effects via various pathways, including Pathways in cancer, Lipid and Atherosclerosis, Fluid shear stress and atherosclerosis, as well as the AGE-RAGE signaling pathway. Lastly, this study provides a valuable reference for further exploration of the therapeutic mechanisms of the Chufeng Qingpi Decoction in the treatment of schistosomiasis.

## Data Availability

The original contributions presented in the study are included in the article/**Supplementary Material**. Further inquiries can be directed to the corresponding authors.

## Supplementary material

Supplementary Figure 1 Interaction plan of four compounds.

Supplementary Figure 2 The overall binding free energy of the compound composed of TP53 and the other four small molecules.

## Glossary

CQD: Chufeng Qingpi Decoction
PPI: Protein-Protein Interaction
GO: Gene Ontology
KEGG: Kyoto Encyclopedia of Genes and Genomes
LASSO: Least Absolute Shrinkage and Selection Operator
RF: Random Forest
SVM-RFE: Support Vector Machine with Recursive Feature Elimination
PTGS2: Prostaglandin-endoperoxide synthase 2
TNF: Tumor Necrosis Factor
TGFB1: Transforming Growth Factor Beta 1
BCL2: Transforming Growth Factor Beta 1
TP53: Tumor Protein 53
IL10: Interleukin-10
JUN: Jun Proto-Oncogene, AP-1 Transcription Factor Subunit
MMP2: Matrix Metalloproteinase-2
IL1B: Interleukin-1 β
MYC: Myelocytomatosis Oncogene
OB: Oral Bioavailability
DL: Drug Likeness
BC: Betweenness Centrality
CC: Closeness Centrality
BP: Biological Process
CC: Cellular Component
MF: Molecular Function
RMSD: Root Mean Square Deviation
TIMP-1: Tissue Inhibitor of Metallo Proteinase 1
