# Supplementary figures and images for: Combining network pharmacology, machine learning, molecular docking and molecular dynamic to explore the mechanism of Chufeng Qingpi decoction in treating schistosomiasis
Source: Front Cell Infect Microbiol. 2024 Sep 6;14:1453529. doi: 10.3389/fcimb.2024.1453529 (PMC11413488; doi:10.3389/fcimb.2024.1453529)

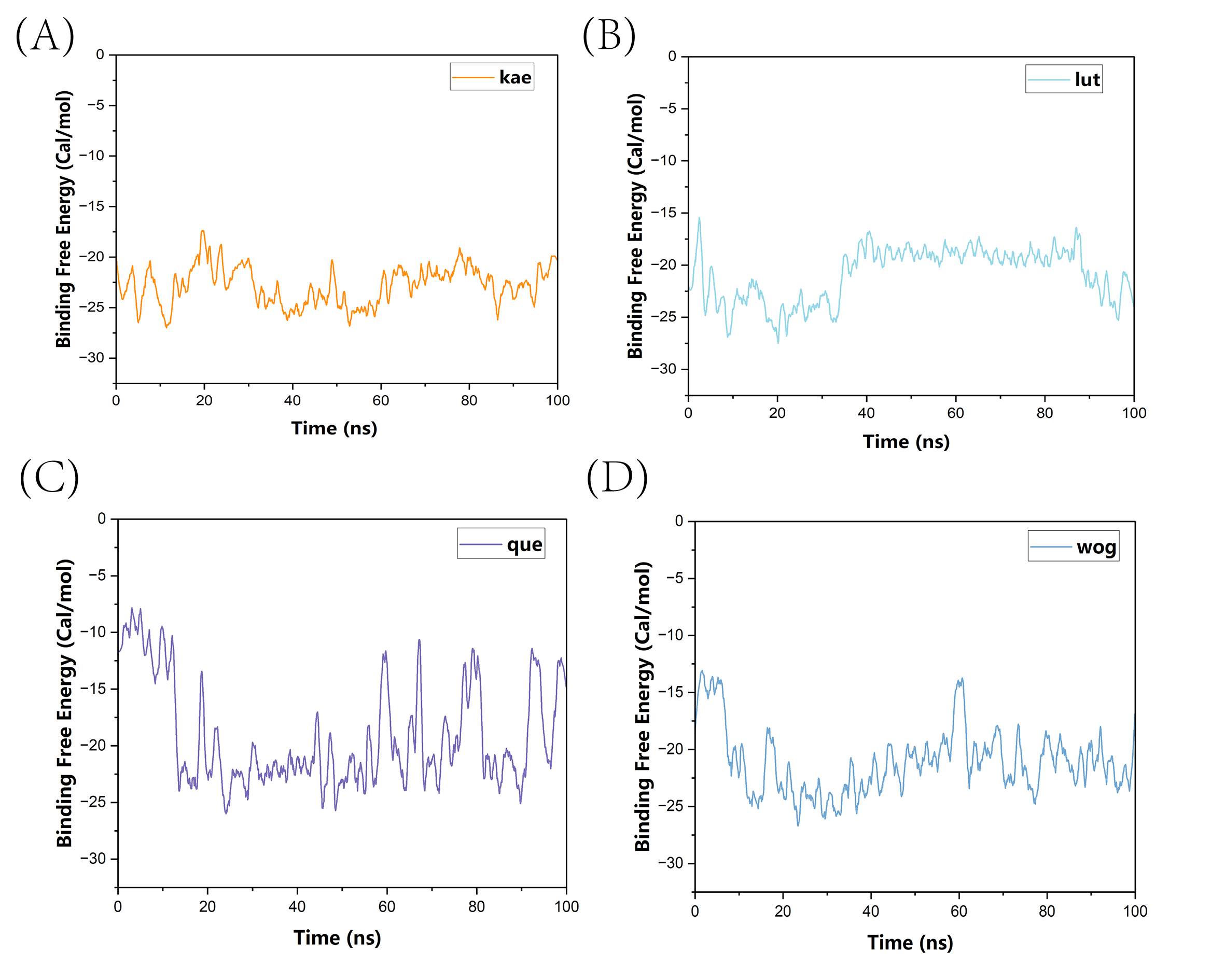

Supplement: Supplementary Figure 1 — Interaction plan of four compounds. [file Image1.tif]

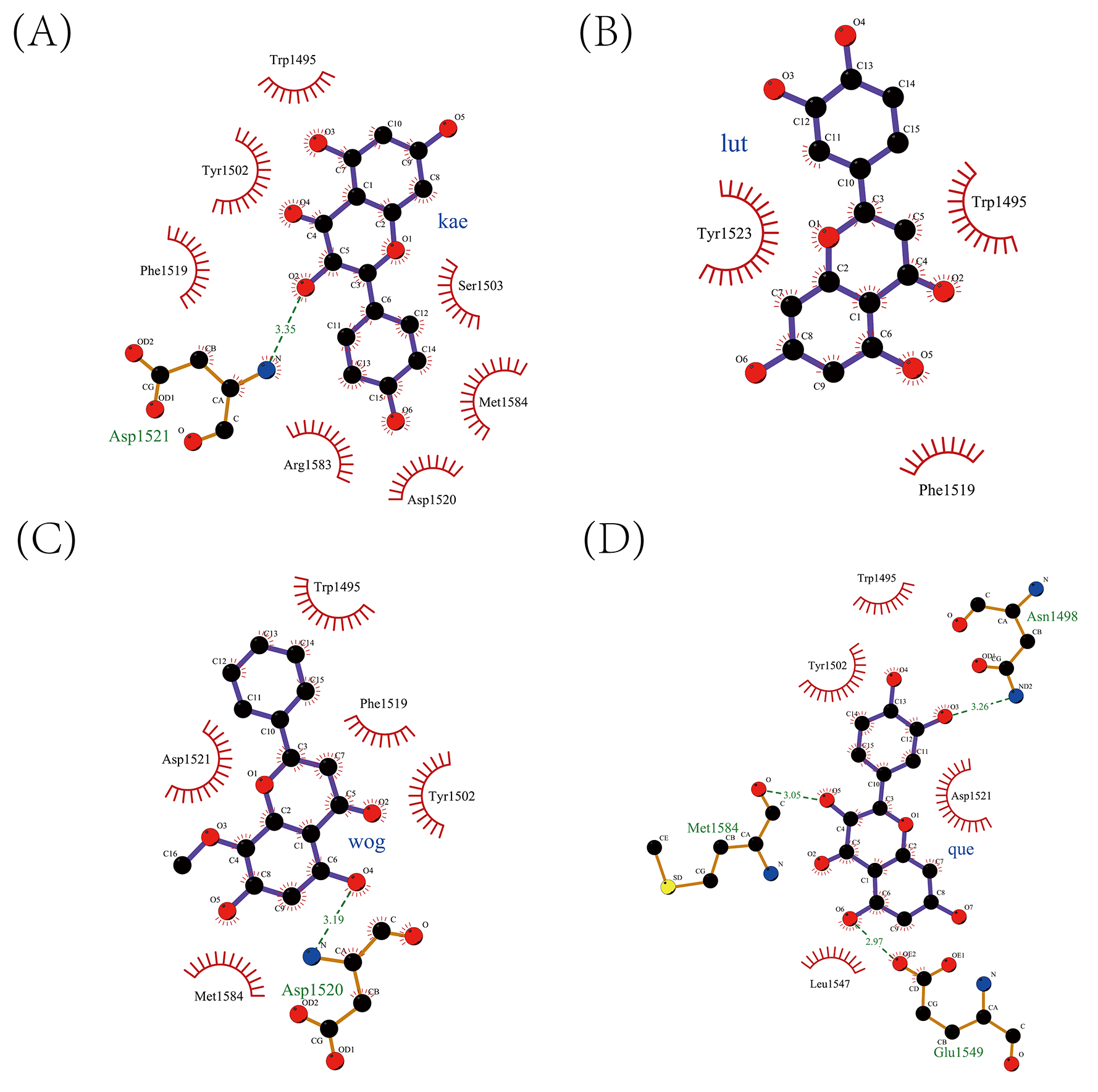

Supplement: Supplementary Figure 2 — The overall binding free energy of the compound composed of TP53 and the other four small molecules. [file Image2.tif]
