# Supplementary material for: Combining network pharmacology, machine learning, molecular docking and molecular dynamic to explore the mechanism of Chufeng Qingpi decoction in treating schistosomiasis
Source: Front Cell Infect Microbiol. 2024 Sep 6;14:1453529. doi: 10.3389/fcimb.2024.1453529 (PMC11413488; doi:10.3389/fcimb.2024.1453529)
Supplement: Supplementary file 3 [file Table1.docx]

| **SUPPLEMENTARY TABLE 1 \|** Conversion between component numbers and MOLIDs. | | | |
| --- | --- | --- | --- |
| **Component numbers** | **MOLIDs** | **Molecule Name** | **Molecule Structure** |
| A1 | MOL000359 | sitosterol | 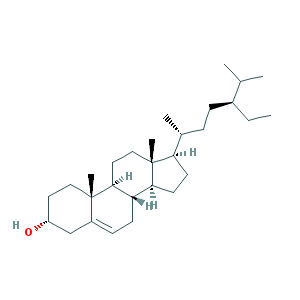 |
| B1 | MOL000358 | beta-sitosterol | 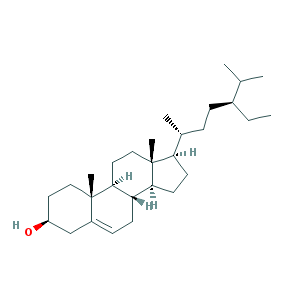 |
| C1 | MOL000449 | Stigmasterol | 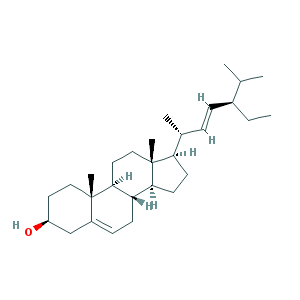 |
| D1 | MOL000173 | wogonin | 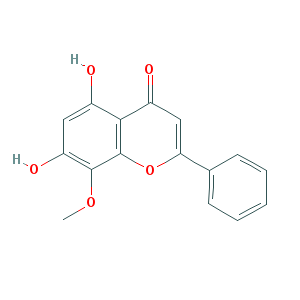 |
| E1 | MOL002897 | epiberberine | 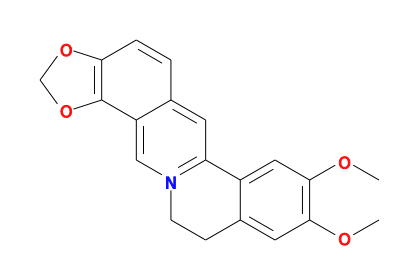 |
| F1 | MOL001458 | coptisine | 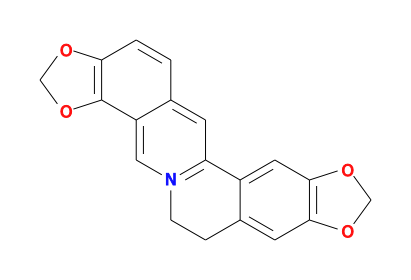 |
| G1 | MOL000098 | quercetin | 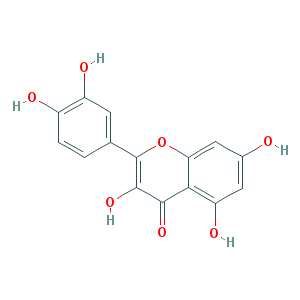 |
| H1 | MOL001689 | acacetin | 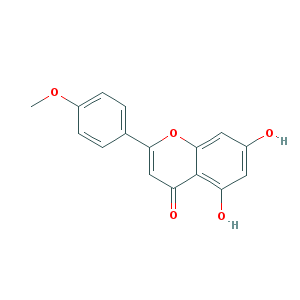 |
| I1 | MOL000006 | luteolin | 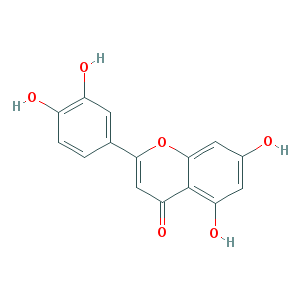 |
| J1 | MOL000422 | kaempferol | 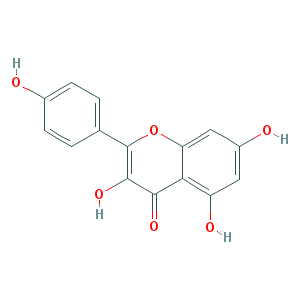 |
| CP1 | MOL004328 | naringenin | 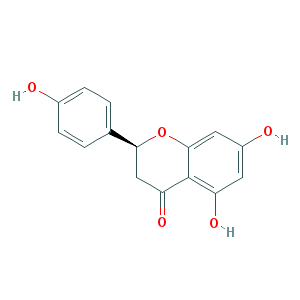 |
| CP2 | MOL005100 | 5,7-dihydroxy-2-(3-hydroxy-4-methoxyphenyl)chroman-4-one | 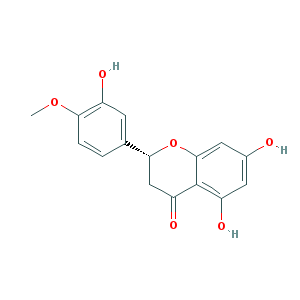 |
| CP3 | MOL005815 | Citromitin | 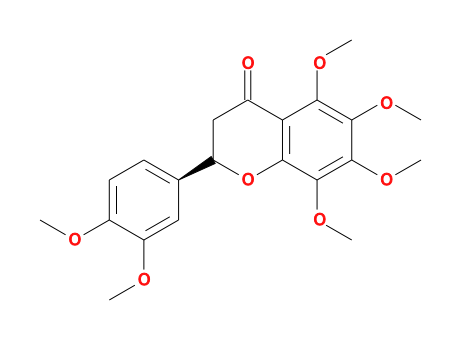 |
| CP4 | MOL005828 | nobiletin | 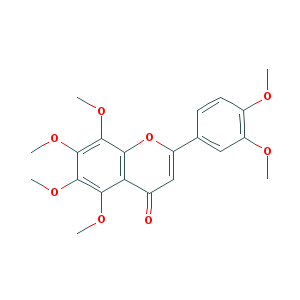 |
| DH1 | MOL002235 | EUPATIN | 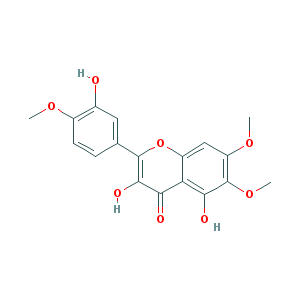 |
| DH2 | MOL002259 | Physciondiglucoside | 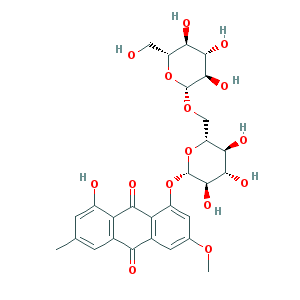 |
| DH3 | MOL002268 | rhein | 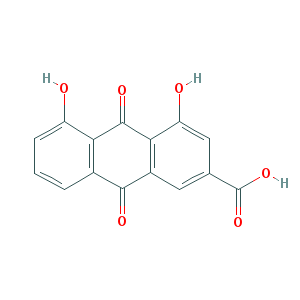 |
| DH4 | MOL002280 | Torachrysone-8-O-beta-D-(6'-oxayl)-glucoside | 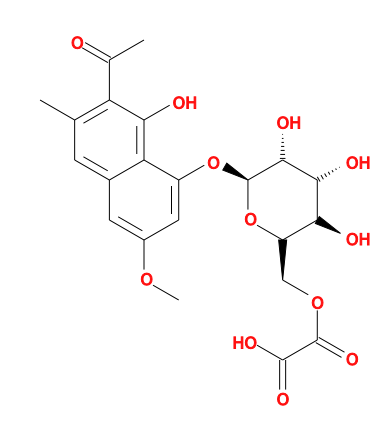 |
| DH5 | MOL002281 | Toralactone | 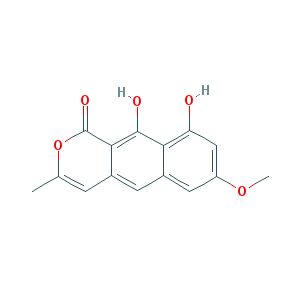 |
| DH6 | MOL002288 | Emodin-1-O-beta-D-glucopyranoside | 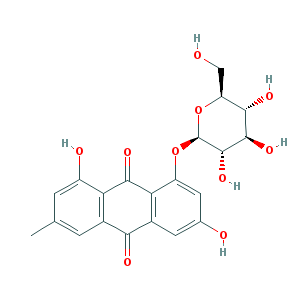 |
| DH7 | MOL002297 | Daucosterol_qt | 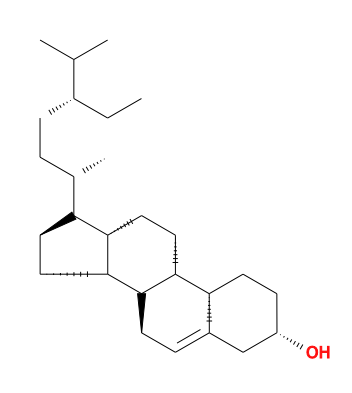 |
| DH8 | MOL000471 | aloe-emodin | 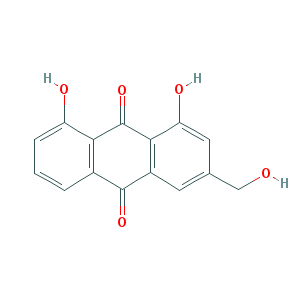 |
| DH9 | MOL000096 | (-)-catechin | 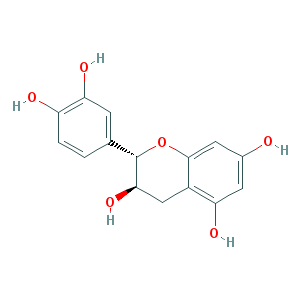 |
| FF1 | MOL000011 | (2R,3R)-3-(4-hydroxy-3-methoxy-phenyl)-5-methoxy-2-methylol-2,3-dihydropyrano[5,6-h][1,4]benzodioxin-9-one | 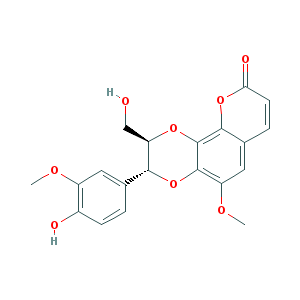 |
| FF2 | MOL011730 | 11-hydroxy-sec-o-beta-d-glucosylhamaudol_qt | 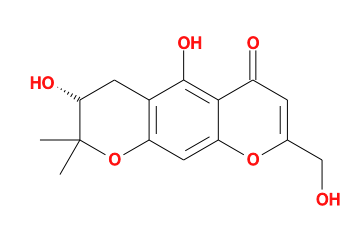 |
| FF3 | MOL011732 | anomalin | 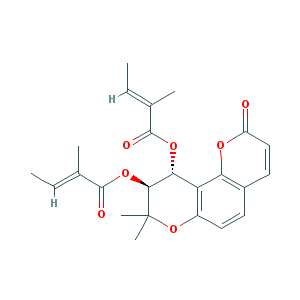 |
| FF4 | MOL011737 | divaricatacid | 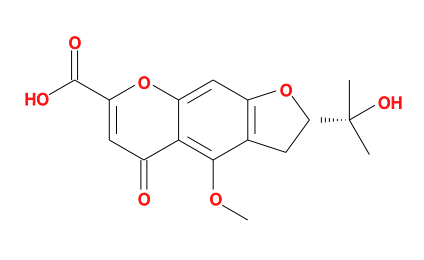 |
| FF5 | MOL011740 | divaricatol | 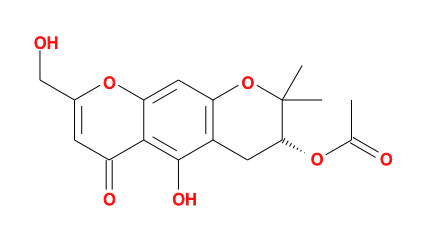 |
| FF6 | MOL001941 | Ammidin | 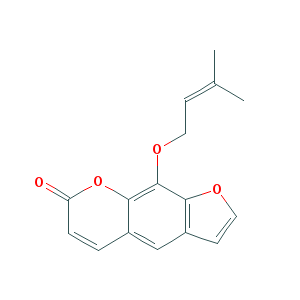 |
| FF7 | MOL011747 | ledebouriellol | 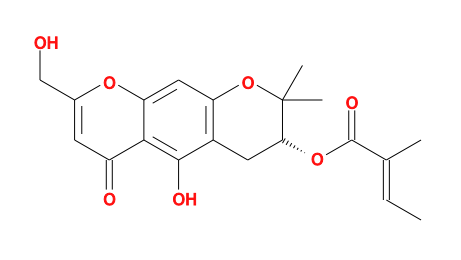 |
| FF8 | MOL011749 | phelloptorin | 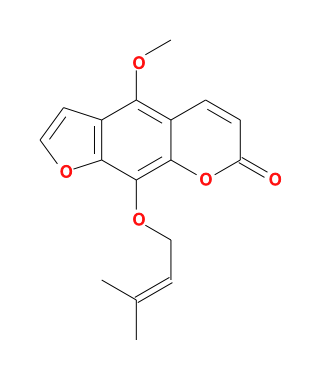 |
| FF9 | MOL011753 | 5-O-Methylvisamminol | 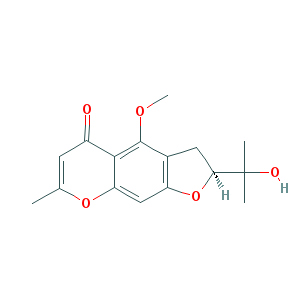 |
| FF10 | MOL002644 | Phellopterin | 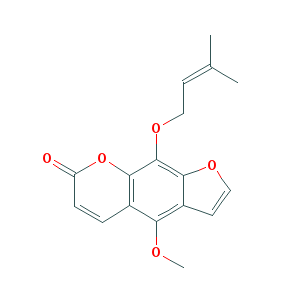 |
| FF11 | MOL001494 | Mandenol | 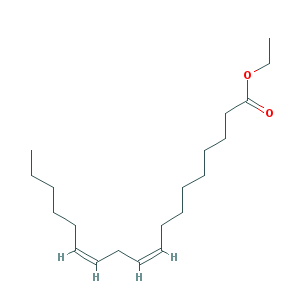 |
| FF12 | MOL001942 | isoimperatorin | 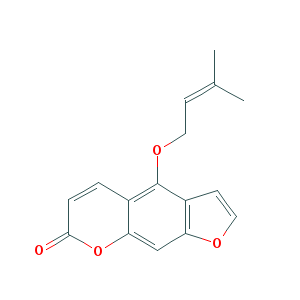 |
| FF13 | MOL003588 | Prangenidin | 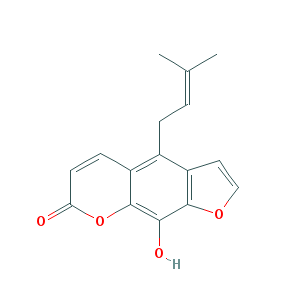 |
| FF14 | MOL007514 | methyl icosa-11,14-dienoate | 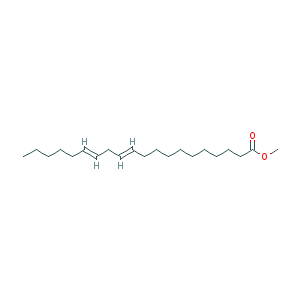 |
| FF15 | MOL013077 | Decursin | 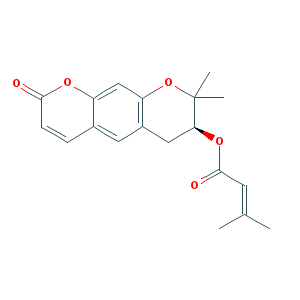 |
| HL1 | MOL001454 | berberine | 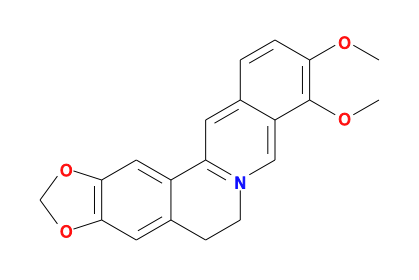 |
| HL2 | MOL002894 | berberrubine | 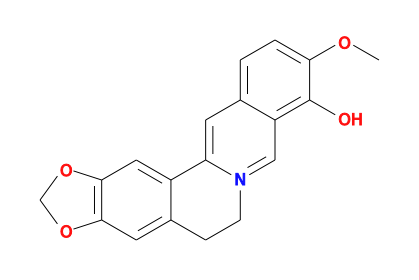 |
| HL3 | MOL002903 | (R)-Canadine | 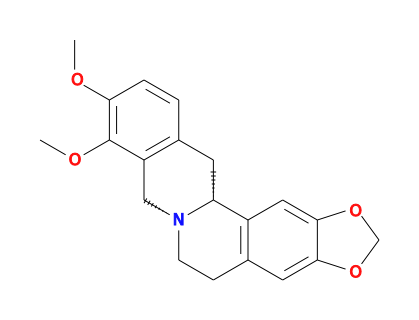 |
| HL4 | MOL002904 | Berlambine | 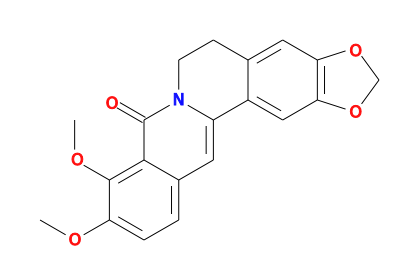 |
| HL5 | MOL002907 | Corchoroside A_qt | 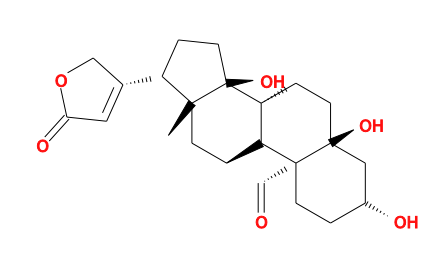 |
| HL6 | MOL000622 | Magnograndiolide | 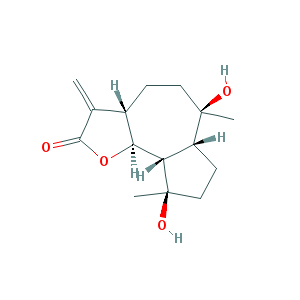 |
| HL7 | MOL000785 | palmatine | 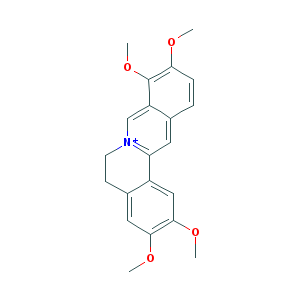 |
| HL8 | MOL002668 | Worenine | 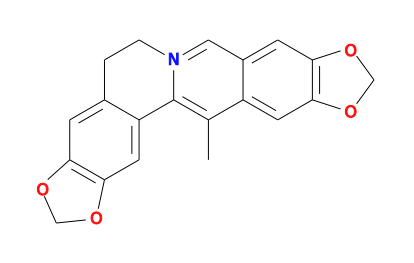 |
| HQ1 | MOL000228 | (2R)-7-hydroxy-5-methoxy-2-phenylchroman-4-one | 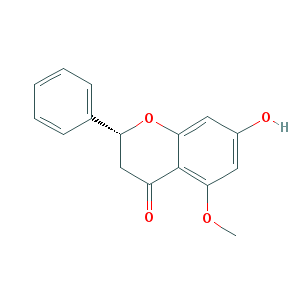 |
| HQ10 | MOL002927 | Skullcapflavone II | 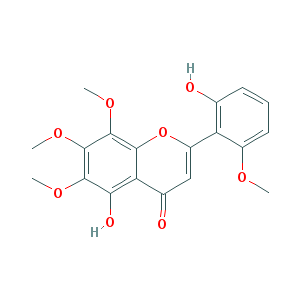 |
| HQ11 | MOL002928 | oroxylin a | 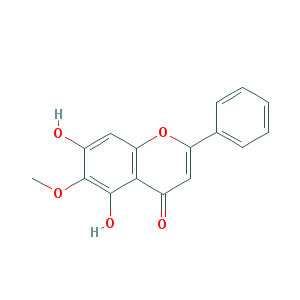 |
| HQ12 | MOL002932 | Panicolin | 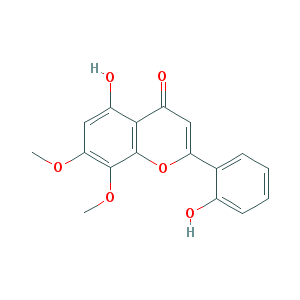 |
| HQ13 | MOL002933 | 5,7,4'-Trihydroxy-8-methoxyflavone | 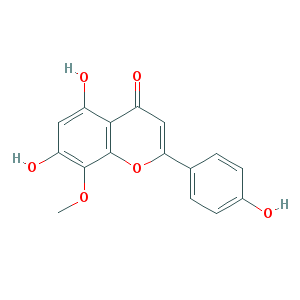 |
| HQ14 | MOL002934 | NEOBAICALEIN | 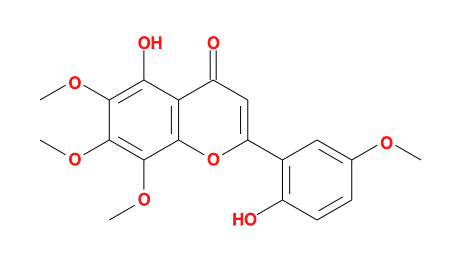 |
| HQ15 | MOL002937 | DIHYDROOROXYLIN | 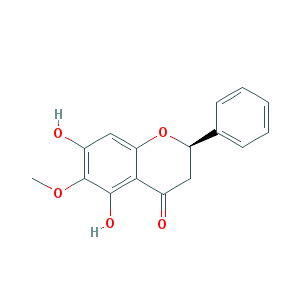 |
| HQ16 | MOL000525 | Norwogonin | 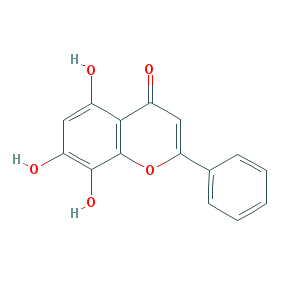 |
| HQ17 | MOL000552 | 5,2'-Dihydroxy-6,7,8-trimethoxyflavone | 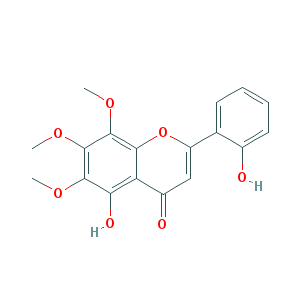 |
| HQ18 | MOL000073 | ent-Epicatechin | 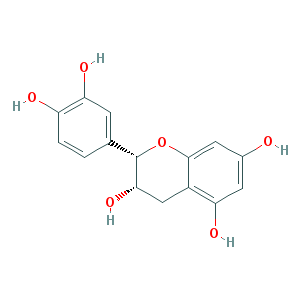 |
| HQ19 | MOL001490 | bis[(2S)-2-ethylhexyl] benzene-1,2-dicarboxylate | 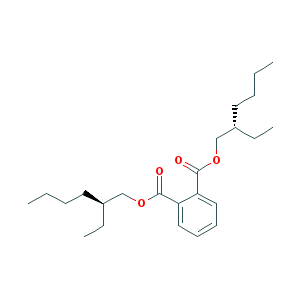 |
| HQ2 | MOL002714 | baicalein | 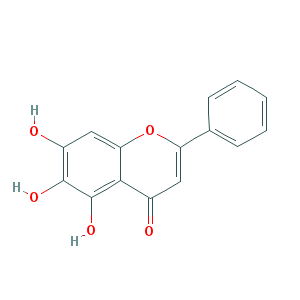 |
| HQ20 | MOL002879 | Diop | 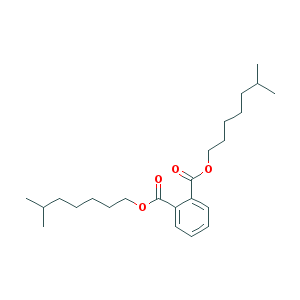 |
| HQ21 | MOL008206 | Moslosooflavone | 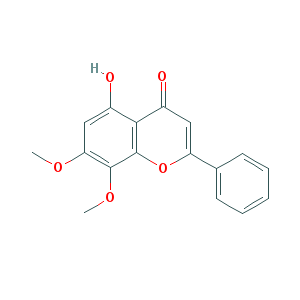 |
| HQ22 | MOL010415 | 11,13-Eicosadienoic acid, methyl ester | 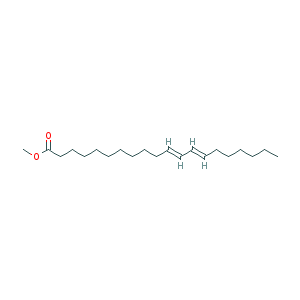 |
| HQ23 | MOL012245 | 5,7,4'-trihydroxy-6-methoxyflavanone | 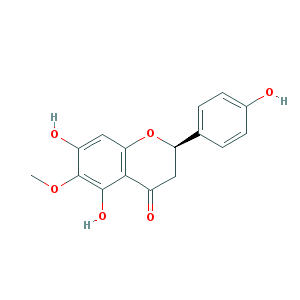 |
| HQ24 | MOL012246 | 5,7,4'-trihydroxy-8-methoxyflavanone | 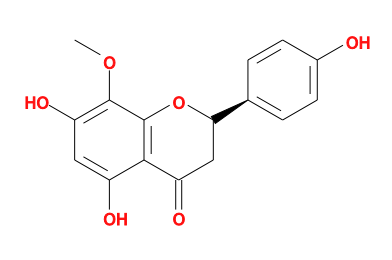 |
| HQ25 | MOL012266 | rivularin | 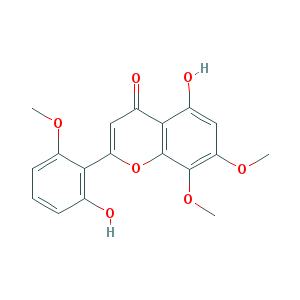 |
| HQ3 | MOL002909 | 5,7,2,5-tetrahydroxy-8,6-dimethoxyflavone | 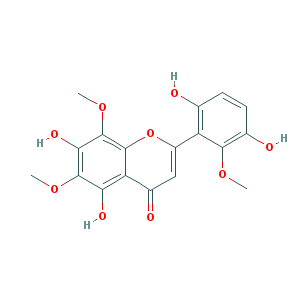 |
| HQ4 | MOL002910 | Carthamidin | 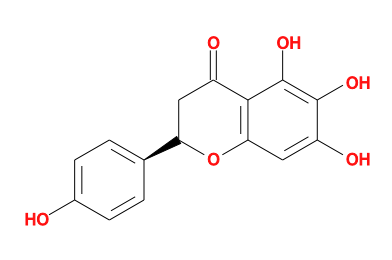 |
| HQ5 | MOL002913 | Dihydrobaicalin_qt | 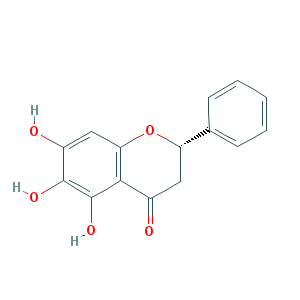 |
| HQ6 | MOL002914 | Eriodyctiol (flavanone) | 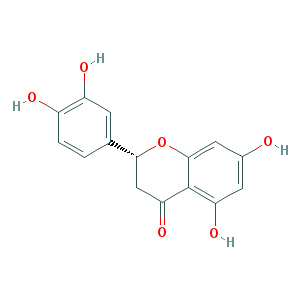 |
| HQ7 | MOL002915 | Salvigenin | 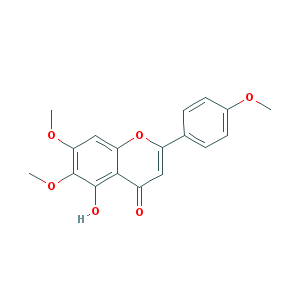 |
| HQ8 | MOL002917 | 5,2',6'-Trihydroxy-7,8-dimethoxyflavone | 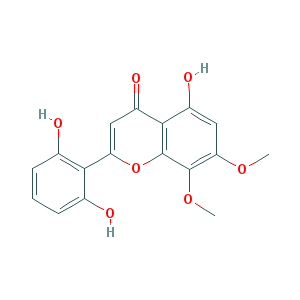 |
| HQ9 | MOL002925 | 5,7,2',6'-Tetrahydroxyflavone | 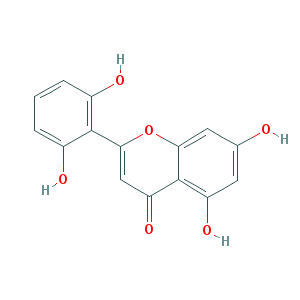 |
| JJS1 | MOL002341 | Hesperetin | 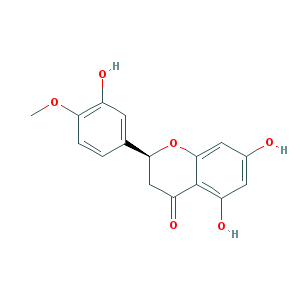 |
| JJS2 | MOL005842 | Pectolinarigenin | 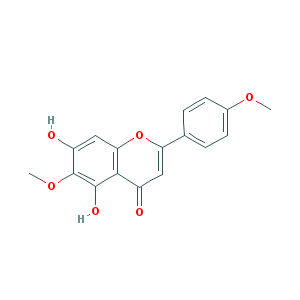 |
| JJS3 | MOL011849 | Schizonepetoside B | 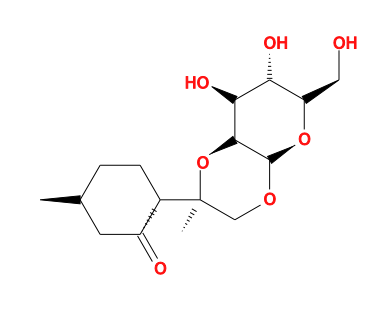 |
| JG1 | MOL001689 | acacetin | 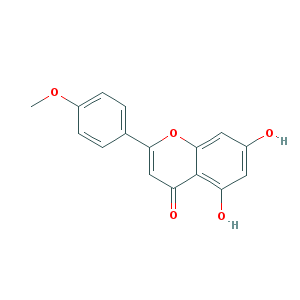 |
| JG2 | MOL004355 | Spinasterol | 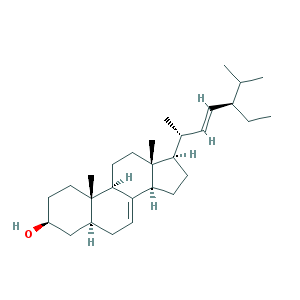 |
| JG3 | MOL004580 | cis-Dihydroquercetin | 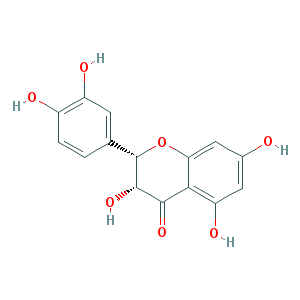 |
| LQ1 | MOL003283 | (2R,3R,4S)-4-(4-hydroxy-3-methoxy-phenyl)-7-methoxy-2,3-dimethylol-tetralin-6-ol | 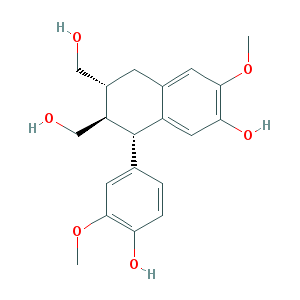 |
| LQ10 | MOL003330 | (-)-Phillygenin | 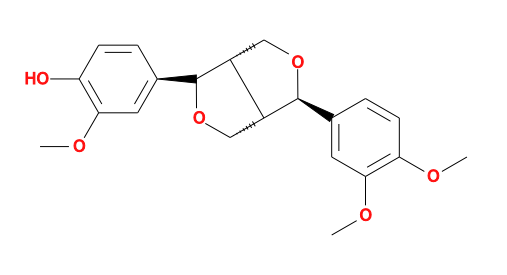 |
| LQ11 | MOL003347 | hyperforin | 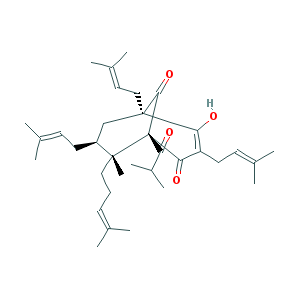 |
| LQ12 | MOL003370 | Onjixanthone I | 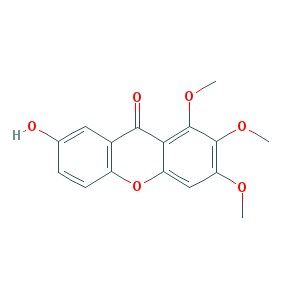 |
| LQ13 | MOL000522 | arctiin | 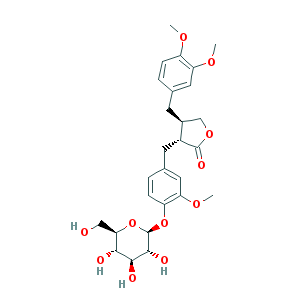 |
| LQ14 | MOL000791 | bicuculline | 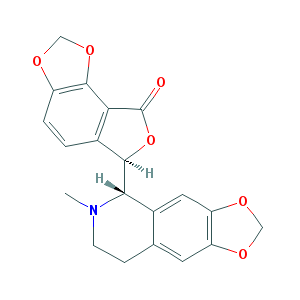 |
| LQ2 | MOL003290 | (3R,4R)-3,4-bis[(3,4-dimethoxyphenyl)methyl]oxolan-2-one | 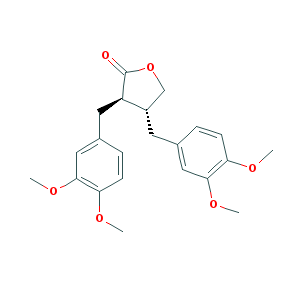 |
| LQ3 | MOL003295 | (+)-pinoresinol monomethyl ether | 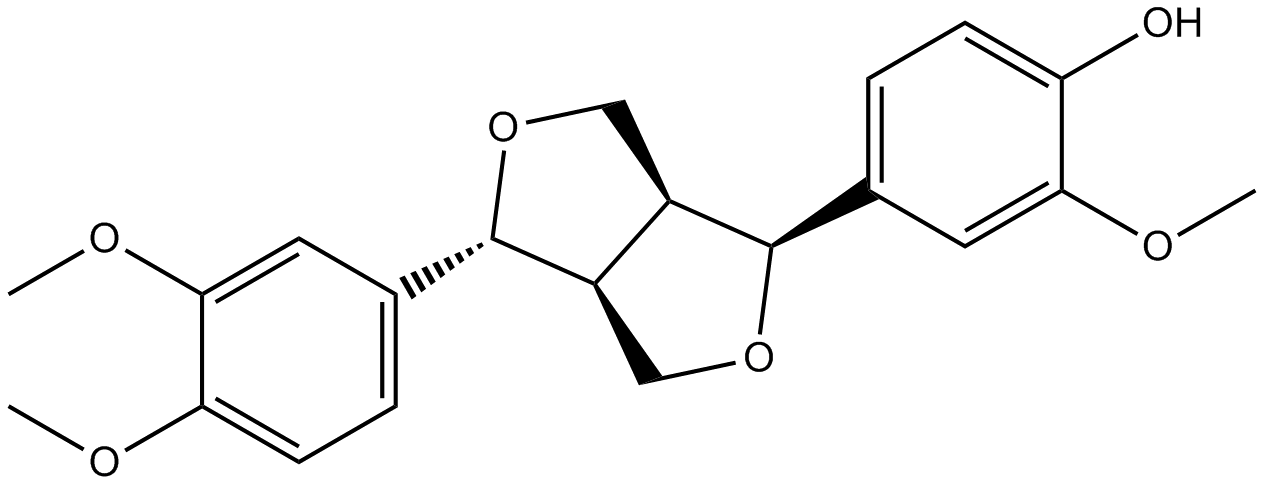 |
| LQ4 | MOL003305 | PHILLYRIN | 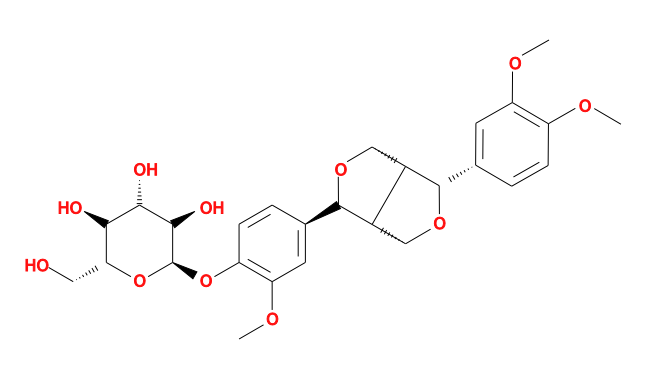 |
| LQ5 | MOL003306 | ACon1_001697 | 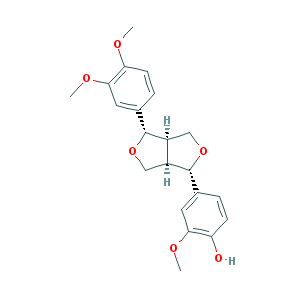 |
| LQ6 | MOL003308 | (+)-pinoresinol monomethyl ether-4-D-beta-glucoside_qt | 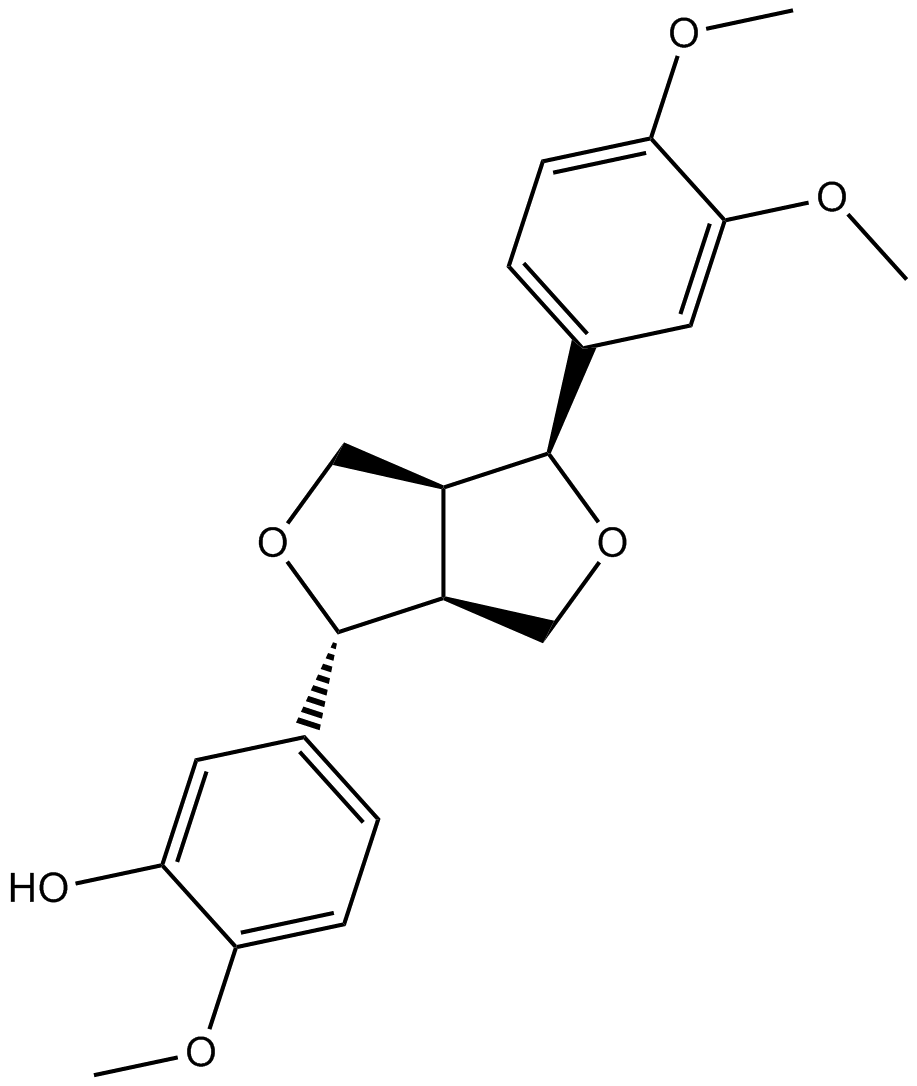 |
| LQ7 | MOL003315 | 3beta-Acetyl-20,25-epoxydammarane-24alpha-ol | 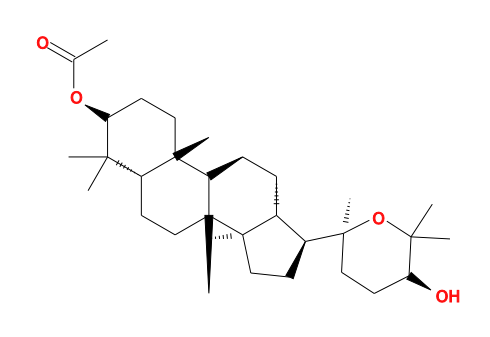 |
| LQ8 | MOL000211 | Mairin | 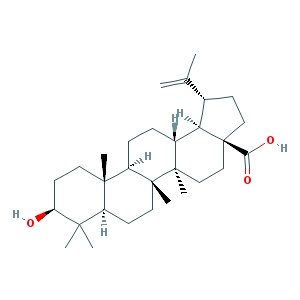 |
| LQ9 | MOL003322 | FORSYTHINOL | 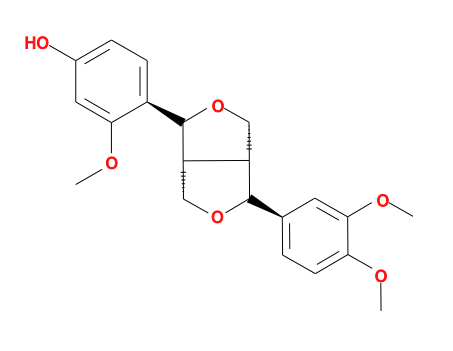 |
| XS1 | MOL002222 | sugiol | 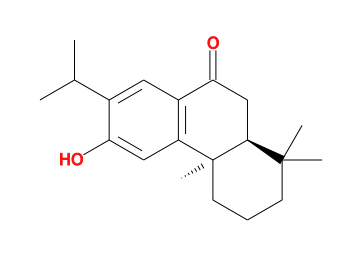 |
| XS2 | MOL007658 | 14-deoxy-12(R)-sulfoandrographolide | 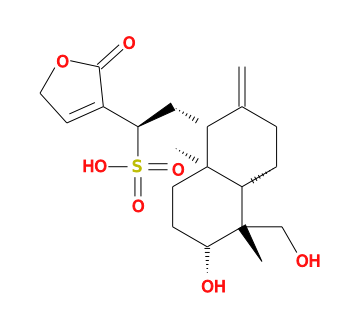 |
| XS3 | MOL007662 | harpagoside_qt | 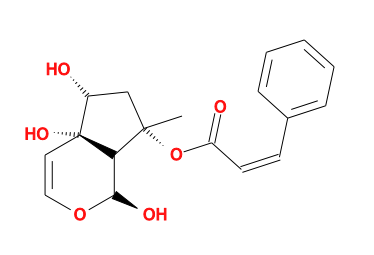 |
| ZM9 | MOL000546 | diosgenin | 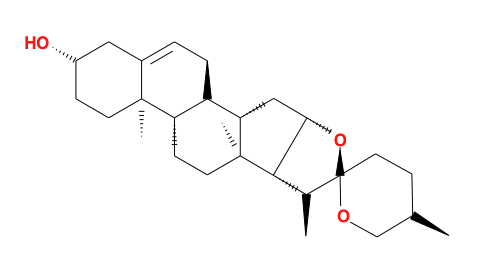 |
| ZM8 | MOL000483 | (Z)-3-(4-hydroxy-3-methoxy-phenyl)-N-[2-(4-hydroxyphenyl)ethyl]acrylamide | 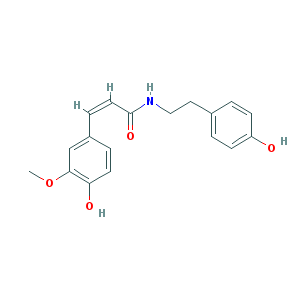 |
| ZM7 | MOL004540 | Anemarsaponin C_qt | 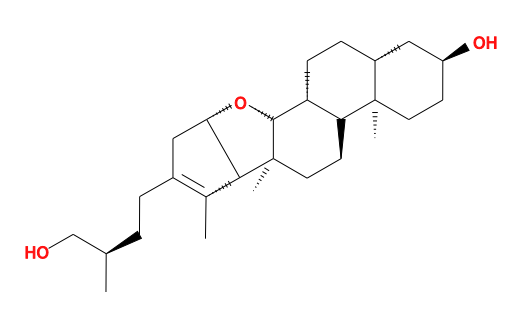 |
| ZM6 | MOL004528 | Icariin I | 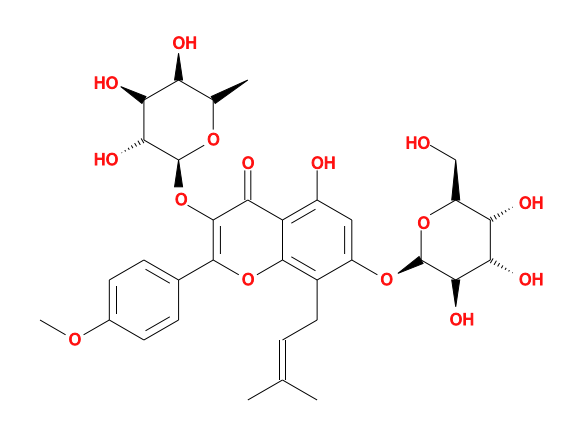 |
| ZM5 | MOL004514 | Timosaponin B III_qt | 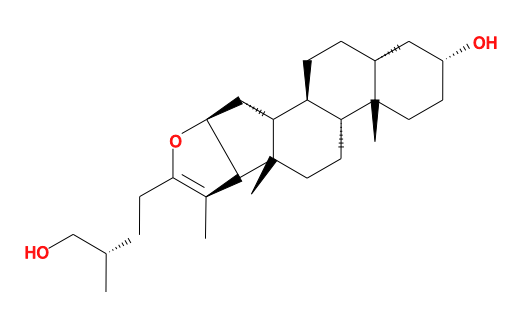 |
| ZM4 | MOL004497 | Hippeastrine | 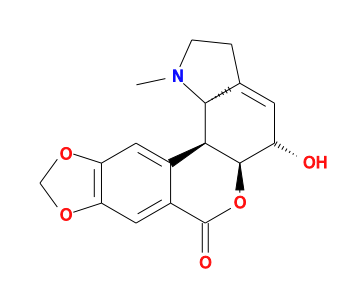 |
| ZM3 | MOL004489 | Anemarsaponin F_qt |  |
| ZM2 | MOL004373 | Anhydroicaritin |  |
| ZM10 | MOL000631 | coumaroyltyramine |  |
| ZM1 | MOL001677 | asperglaucide |  |
